# Rice (*Oryza sativa*) TIR1 and 5′adamantyl-IAA Significantly Improve the Auxin-Inducible Degron System in *Schizosaccharomyces pombe*

**DOI:** 10.3390/genes12060882

**Published:** 2021-06-08

**Authors:** Adam T. Watson, Storm Hassell-Hart, John Spencer, Antony M. Carr

**Affiliations:** 1 Genome Damage and Stability Centre, School of Life Sciences, University of Sussex, Brighton BN1 9RQ, UK; a.t.watson@sussex.ac.uk; 2 Department of Chemistry, School of Life Sciences, University of Sussex, Brighton BN1 9QJ, UK; S.Hassell-Hart@sussex.ac.uk (S.H.-H.); J.Spencer@sussex.ac.uk (J.S.)

**Keywords:** AID, AID2, degron, auxin, fission yeast, protein degradation

## Abstract

The auxin-inducible degron (AID) system is a powerful tool to induce targeted degradation of proteins in eukaryotic model organisms. The efficiency of the existing *Schizosaccharomyces pombe* AID system is limited due to the fusion of the F-box protein TIR1 protein to the SCF component, Skp1 (Skp1-TIR1). Here, we report an improved AID system for *S. pombe* that uses the TIR1 from *Oryza sativa* (OsTIR1) not fused to Skp1. Furthermore, we demonstrate that degradation efficiency can be improved by pairing an OsTIR1 auxin-binding site mutant, OsTIR1^F74A^, with an auxin analogue, 5′adamantyl-IAA (AID2). We provide evidence for the enhanced functionality of the OsTIR1 AID and AID2 systems by application to the essential DNA replication factor Mcm4 and to a non-essential recombination protein, Rad52. Unlike AID, no detectable auxin-independent depletion of AID-tagged proteins was observed using AID2.

## 1. Introduction

The auxin-inducible degron (AID) is an effective method for the rapid depletion of target proteins, allowing control of protein expression and the study of protein function in vivo [1]. In plants, the physiological role of auxin is to regulate growth and development by mediating the interaction of the F-box protein transport inhibitor response 1 (TIR1) with the auxin/indole-3-acetic acid (AUX/IAA) family of transcriptional regulators [2]. TIR1 is part of the E3 ubiquitin ligase SCF-TIR1 (Skp/Cullin/F-box) complex that recruits an E2 ubiquitin-conjugating enzyme and polyubiquitinates AUX/IAA proteins, targeting them for degradation by the proteasome.

**Figure 1:**
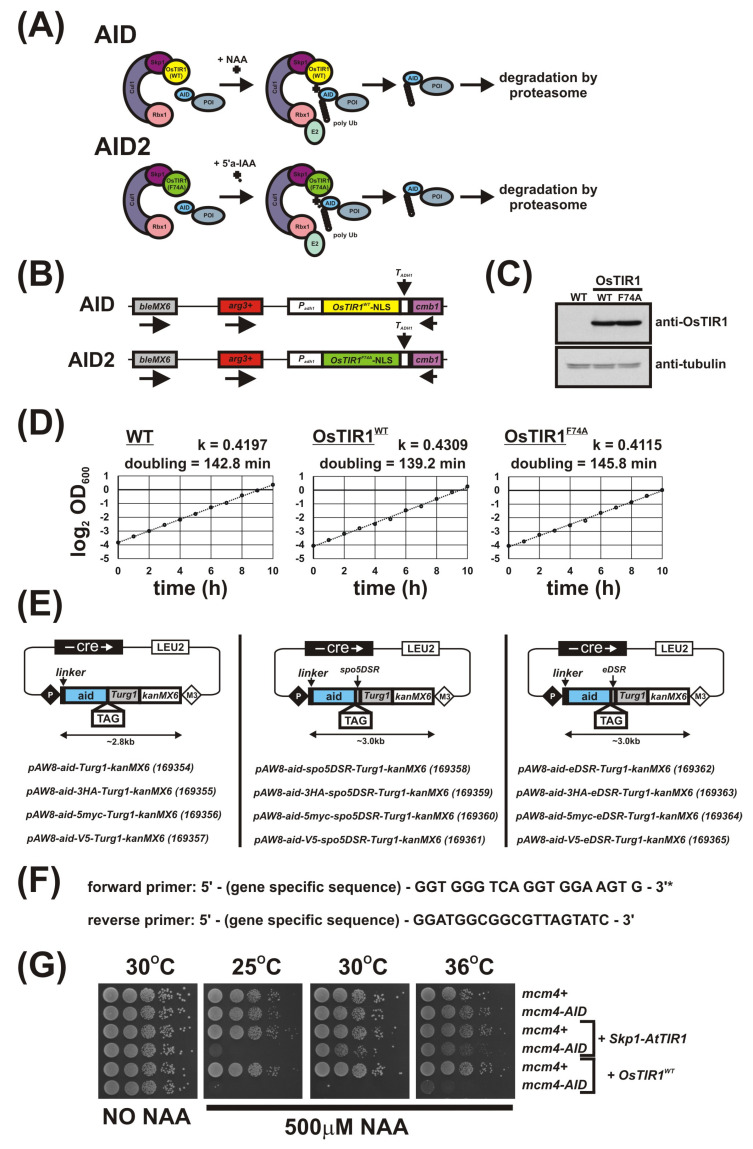
AID/OsTIR1^WT^ and AID2/OsTIR1^F74A^ systems in *S. pombe.* (**A**) Schematic illustration showing AID/OsTIR1^WT^ and AID2/OsTIR1^F74A^ systems. AID: As the Skp1, Cullin and F-box protein complex (SCF) is highly conserved among eukaryotes, exogenously expressed plant F-box protein OsTIR1 can form a functional SCF complex in *S. pombe*. The protein of interest (POI) is fused to the AID degron (AID). The synthetic auxin 1-naphthaleneacetic acid (NAA) binds OsTIR1 and promotes interaction between OsTIR1 and the AID degron. SCF-OsTIR1 acts as an E3 ubiquitin ligase to recruit an E2 ubiquitin-conjugating enzyme, resulting in the poly-ubiquitination of the AID tag. Finally, the POI is degraded by the proteasome. AID2: The process is analogous to AID, but pairs the OsTIR1 auxin-binding site mutant F74A and the 5’-substituted auxin analogue 5′adamantyl-indole-3-acetic acid (5′a-IAA). (**B**) Schematic illustration showing the *arg3-D4* loci engineered to constitutively express OsTIR1^WT^ or OsTIR1^F74A^ (NLS: nuclear localisation signal). (**C**) Representative immunoblot on total protein extract from wild-type^WT^ (AW279), *OsTIR1^WT^* (AW1762) and *OsTIR1^F74A^* (AW1703) cells detected using anti-OsTIR1 antibody. Anti-tubulin was used as a loading control. (**D**) Representative growth curves of isogenic wild-type (WT—(AW279)), *OsTIR1^WT^* (AW1762) and *OsTIR1^F74A^* (AW1703) cells. Cells were grown in YE media at 30 °C with constant shaking (180 rpm). Optical density (OD) was measured at 1h intervals for total 10 h. Timeseries of log 2-transformed OD_600_ measurements are presented. Black lines represent linear regression models and are the mean of two technical repeats. Slopes of linear regression models (k) and calculated doubling times are indicated. (**E**) Plasmids to C-terminally AID-tag a protein of interest (linker: flexible 10 amino acid linker (Gly-Gly-Ser-Gly-Gly-Ser-Gly-Ser-Gly-Ala), *spo5*DSR: *spo5* Determinant of Selective Removal element, eDSR: enhanced *spo5*DSR element, TAG: epitope tag, number in parenthesis after plasmid name: Addgene ID). (**F**) Primer sequences to amplify *aid-(TAG)-(DSR)-Turg1-kanMX6* construct. * Reading frame for tag is indicated. (**G**) Isogenic strains with the indicated genotypes (*mcm4+* (AW279), *mcm4-AID* (AW1893), *Skp1-AtTIR1* (AW1942), *mcm4-AID Skp1-AtTIR1* (AW1949), *OsTIR1^WT^* (AW1762), *mcm4-AID, OsTIR1^WT^* (AW1923)) were spotted onto YEA plates and plates containing 500 μM NAA. Plates were grown at the indicated temperatures for 3 days. The experiment was repeated and similar results were obtained.

This degradation pathway can be transferred to non-plant organisms by (1) exogenously expressing the auxin receptor F-box protein, TIR1 and (2) the fusion of an AUX/IAA auxin-inducible degron (AID) to the protein of interest. As the other components of the SCF complex are highly conserved, these non-plant cells can be treated with auxin to induce proteasomal degradation of the AID-tagged protein (Figure 1A). In current AID systems, *Oryza sativa* TIR1 (OsTIR1) is the most commonly used auxin receptor F-box protein and is used in combination with AID tags derived from the *Arabidopsis thaliana* IAA17 protein [1,3,4,5,6,7,8,9]. However, the fission yeast AID system developed by Kanke et al. [10] uses a fusion of the *A. thaliana* TIR1 (AtTIR1) to the F-box-interacting component of the SCF, Skp1 (Skp1-AtTIR1, system termed ‘*i*-AID’). Overexpression of the Skp1-AtTIR1 fusion using the constitutive alcohol dehydrogenase *adh1* promoter severely inhibited cell growth, so expression levels were reduced using an attenuated version of the *adh1* promoter, *Padh15* (*Padh15-skp1-AtTIR1*) [11]. Despite *i*-AID causing severe replication defects and cell cycle arrest in *mcm4-AID* cells, biologically significant amounts of other target proteins were shown to remain after depletion. The system was improved by exogenous expression of *Padh15-skp1-AtTIR1* and *Padh15-skp1-OsTIR1* (termed ‘*double TIR1′*). *Double TIR1* combined with transcription repression of the target protein (termed ‘*off*-AID’) was used to effectively deplete Pol1 and Cdc45 [10]. The *off*-AID system has been used to efficiently regulate various other proteins including Mcm10 [12], Cdc20 [13], Cnd3 and Smc2 [14] and Bqt1 [15].

A common feature of AID systems is the chronic, auxin-independent proteasome-mediated degradation of AID-tagged proteins in cells constitutively expressing TIR1. To circumvent this basal degradation level and its inherent problems, expression of TIR1 is often transcriptionally regulated. Alternatively, another component of the plants native auxin signalling machinery, an auxin-response transcription factor (ARF), is co-expressed as this has been shown to allow near-endogenous expression levels of the target protein in the absence of auxin [16].

Through a bump-and-hole strategy, a synthetic auxin–receptor pair involving 5-(3-methoxy-phenyl)-IAA and its complementary auxin-binding pocket mutant AtTIR1^F79G^ was identified and shown to function orthogonally to endogenous auxin signalling [17]. A screen of 5-substituted IAA analogues using a yeast two-hybrid system identified 5′adamantyl-indole-3-acetic acid (5′adamantyl-IAA) which mediates interaction of AtTIR1^F79G^ and IAA3 proteins at a 100-fold lower concentration compared to the natural auxin–TIR1 pair. Following a screen of binding pocket mutants other than glycine, AtTIR1^F79A^ was shown to interact with IAA3 proteins at picomolar concentrations of 5′adamantyl-IAA, 10,000-fold lower than for the auxin–TIR1 pair [18]. The corresponding OsTIR1-binding site mutant (OsTIR1^F74A^) and 5′adamantyl-IAA have recently been shown to function in chicken DT40 cells at 1,000-fold lower ligand concentration compared to conventional AID/IAA-OsTIR1^WT^ and shown to function effectively in mammalian cell lines again at significantly lower ligand concentrations [19]. Using an equivalent bump-and-hole strategy, the synthetic ligand–receptor pair OsTIR1^F74G^ and auxin analogue 5-phenyl-indole-3-acetic acid (5-Ph-IAA) were shown to interact at nanomolar concentrations and have been used to rapidly and efficiently deplete mAID-fused proteins in budding yeast, mammalian cells and mice. This modified system was termed AID version 2 (AID2) [6]. The kinetics of protein depletion were quicker when compared to the original AID system and depletion could be induced with several hundred-fold lower ligand concentration. Moreover, no detectable basal degradation was observed using AID2.

In fission yeast, a mechanism for the selective removal of meiosis-specific mRNAs in mitotic cells has been characterised. The removal involves the YTH domain-containing protein Mmi1 [20], which binds meiosis-specific mRNAs containing Determinant of Selective Removal (DSR) sequences, usually located at the 3′ end of the transcript. Mmi1 is only functional in mitotic, but not meiotic, cells and greatly increases transcript turnover by directing DSR-containing transcripts to the nuclear exosomes for degradation [20,21]. By deletion analysis, the DSR elements of *ssm4*, *rec8* and *spo5* mRNAs were identified [20]. Further studies have identified a hexanucleotide motif, U(U/C)AAAC, that is highly enriched in the DSR elements and have shown that tandem repeats of this motif can function as an artificial DSR in heterologous gene systems [22,23].

Here, we describe an improved AID system for fission yeast that overcomes the drawbacks of the original Kanke et al. [10] approach. We constructed two sets of plasmid vector for this study. One set was designed to stably integrate expression constructs at the *arg3-D4* locus [24]. These plasmids were used to insert and constitutively express a genomic copy of the OsTIR1 receptor, OsTIR1^WT^, or the AID2 variant, OsTIR1^F74A^, using the full-strength *S. pombe* alcohol dehydrogenase (*adh1*) promoter. The second set of plasmids was designed to allow AID tagging of the protein of interest at the C-terminus. These plasmids include a series of epitope tag sequences for detection of AID-tagged proteins by commercially available antibodies including HA, Myc and V5. The *spo5*DSR element and an improved engineered version of the *spo5*DSR element, termed ‘eDSR’, are also included as an option that will act to constitutively reduce AID-tagged protein levels. The addition of either the synthetic auxin 1-naphthaleneacetic acid (NAA) (OsTIR1^WT^/AID) or the 5-substituted auxin 5′adamantyl-IAA (OsTIR1^F74A^/AID2), allowed the creation of conditional loss of function mutants of the MCM complex component Mcm4 to be created in cells grown at 30 °C without fusing to Skp1. For AID2, we observed faster depletion kinetics at lower ligand concentrations as compared to AID and no basal degradation of AID-tagged Mcm4. However, for the non-essential recombination protein Rad52, a complete conditional null phenotype was not obtained, despite the presence of DSR sequences: we observed that very low levels of Rad52 activity remained.

## 2. Materials and Methods

### 2.1. Strains and Growth Conditions

Strains used in this work are listed in Table 1. All strains were grown at 30 °C unless stated otherwise. The media composition was as described by Moreno et al. [25]. For selection of G418 and phleomycin-resistant cells, G418 disulphate (Formedium, Hunstanton, UK) or phleomycin (Fisher BioReagents, Waltham, MA, USA) was added to YEA plates at a final concentration of 200 and 30 μg/mL, respectively. Synthetic plant auxin 1-naphthaleneacetic acid (NAA) (Sigma, St. Louis, MO, USA) powder was dissolved in 80% ethanol to the required concentration (0.5 M). The final NAA concentration used was 500 μM. NAA stock was prepared fresh prior to use and unused stock discarded. 5’adamantyl-IAA was dissolved in DMSO to the required concentration (1 mM) and aliquots stored at −20 °C. Growth curve doubling times were calculated using the formula DT = 1/k, where DT stands for doubling time and k represents the slope of linear regression computed from a timeseries of log 2-transformed OD measurements [26].

### 2.2. Cloning and Plasmid Construction

Q5 High-Fidelity 2X Master Mix was used for all PCR reactions in accordance to the manufacturer’s instructions. For all cloning procedures, NEBuilder HiFi DNA Assembly Master Mix was used in accordance to the manufacturer’s instructions. *Escherichia coli* strain DH5α was used for transformations and plasmid isolation. Synthesised gene fragments and custom oligonucleotide primers (Table 2) were ordered from Integrated DNA Technologies.

### 2.3. S. pombe arg3-D4 Integration Expression Vectors

An *Nde*I site contained within the pUC19 backbone was removed by restriction with *Nde*I, blunting the overhangs using Mung Bean Nuclease (New England Biolabs, Ipswich, MA, USA) and religation using T4 DNA ligase (New England Biolabs). The resulting plasmid was termed pUC19*, where * denotes removal of the *Nde*I recognition site from plasmid backbone (CATATG converted to CATG). The *S. pombe arg3-D4* locus was sequenced and the deleted genomic fragment identified. The deleted fragment and approximately 500 bp each side of the deletion site were gene synthesised. Gene synthesis allowed for the removal of unwanted restriction enzymes sites, insertion of a multiple clone site (MCS - *Nhe*I-*Nde*I-*Sph*I-*Xma*I-*Sac*I-*Pst*I-*Sal*I-*Spe*I) and insertion *Not*I restriction enzyme sites for the removal of the linear *arg3* construct. The gene fragment was cloned into *EcoR*I/*Hind*III restricted pUC19* to create MCS- *Sp.arg3+*/pUC19*. The 820 bp of the *S. pombe adh1* promoter sequence (TATA box = TATAAATA) was amplified from total genomic DNA using primers P1 and P2 and the resulting fragment cloned into *Nhe*I/*Nde*I restricted MCS-*Sp.arg3+*/pUC19* to create *P_adh1_*-MCS-*Sp.arg3+*/pUC19*. Approximately 820 bp of the *S. pombe adh15* (TATA box = TAAATATA) and *adh81* (TATA box = AT) promoter sequences were amplified from pKM104 and pKM105 [10] using primers P3 and P4 and the resulting fragment cloned into *Nhe*I/*Nde*I restricted MCS-*Sp.arg3+*/pUC19* to create *P_adh15_*-MCS-*Sp.arg3+*/pUC19* and *P_adh81_*-MCS-*Sp.arg3+*/pUC19*, respectively. Then, 159 bp of the *Saccharomyces cerevisiae ADH1* terminator was amplified from pAW8_TAP [27] using primers P5 and P6 and the fragment cloned into *Sal*I/*Spe*I restricted *P_adh1_*-MCS-*Sp.arg3+*/pUC19*, *P_adh15_*-MCS-*Sp.arg3+*/pUC19* and *P_adh81_*-MCS-*Sp.arg3+*/pUC19* to create *P_adh1_*-MCS-*T_ADH1_*-*Sp.arg3+*/pUC19*, *P_adh15_*-MCS-*T_ADH1_-Sp.arg3+*/pUC19* and *P_adh81_*-MCS-*T_ADH1_*-*Sp.arg3+*/pUC19*, respectively (MCS = *Nde*I-*Xma*I-*Sac*I-*Pst*I-*Sal*I) (Figure S1).

### 2.4. AID-Tagging Constructs

The IAA17 degron tag sequence was amplified from the plasmid template pMK43 [1] using primers P7 and P8 and the fragment cloned into *Sph*I/*Asc*I restricted pAW8_TAP [27], replacing the TAP tag sequence, to create pAW8-aid. Primer P7 encodes a ten amino acid flexible linker sequence (Gly-Gly-Ser-Gly-Gly-Ser-Gly-Ser-Gly-Ala). Primer P8 replaces restriction enzyme site *Asc*I with *Nh*eI. To help identify the presence of the kanMX6 antibiotic selection marker, the plasmid was renamed *pAW8-aid:kanMX6*. 533 bp of the *S. pombe urg1* terminator sequence was amplified from total *S. pombe* genomic DNA using primers P9 and P10 and the product cloned into the *Nhe*I/*Bgl*II restricted *pAW8-aid:kanMX6*, replacing the *S. cerevisiae ADH1* terminator sequence, to create *pAW8-aid-Turg1:kanMX6*. Primer P10 inserted the restriction enzyme recognition site *Xma*I adjacent to the *Nhe*I site. The 157 bp DSR element of the *S. pombe spo5* gene as identified by Harigaya et al. [20] was amplified from pAW8ENdeI-*spo5*DSR [23] using primers P11 and P12 the product cloned into *Nhe*I/*Xm*aI restricted *pAW8-aid-Turg1:kanMX6* to create *pAW8-aid-spo5DSR-Turg1:kanMX6*. Three copies of the human influenza hemagglutinin (HA) protein tag sequence were amplified from plasmid pAW8_3HA [28] using primers P13 and P14 or P13 and P15 the products cloned into *Nhe*I-restricted *pAW8-aid-Turg1:kanMX6* and *Nhe*I-restricted *pAW8-aid-spo5DSR-Turg1:kanMX6*, respectively, to create *pAW8-aid-3HA-Turg1:kanMX6* and *pAW8-aid-3HA-spo5DSR-Turg1:kanMX6*. The forward primer P13 removed the AID-tag stop codon and inserted a small flexible six amino acid linker (Gly-Ala-Gly-Ala-Gly-Ala) between the AID and 3HA tags. Five copies of the human c-myc (Myc) epitope were amplified from plasmid pAW8_13Myc [27] using primers P16 and P17 or P16 and P18 and the resulting fragments cloned into *Nhe*I-restricted *pAW8-aid-Turg1:kanMX6* and *Nhe*I-restricted *pAW8-aid-spo5DSR-Turg1:kanMX6*, respectively, to create *pAW8-aid-5Myc-Turg1:kanMX6* and *pAW8-aid-5Myc-spo5DSR-Turg1:kanMX6*. The forward primer P16 removed the AID-tag stop codon and inserted a small flexible six amino acid linker (Gly-Ala-Gly-Ala-Gly-Ala) between the AID and 5Myc tags. A sequence encoding a single copy of the simian virus 5-derived V5 tag was human codon optimised and gene synthesised and cloned into *Nhe*I-restricted *pAW8-aid-Turg1:kanMX6* to create *pAW8-aid-V5-Turg1:kanMX6*. A second sequence was synthesised allowing cloning into *Nhe*I-restricted *pAW8-aid-spo5DSR-Turg1:kanMX6* to create *pAW8-aid-V5-spo5DSR-Turg1:kanMX6*. The synthesised sequences removed the AID-tag stop codon and inserted a small flexible six amino acid linker (Gly-Ala-Gly-Ala-Gly-Ala) between the AID and V5 tags.

A mutated version of *spo5*DSR element was designed to contain two additional DSR core motifs and was termed ‘eDSR’ (Figure S2). A series of eDSR fragments compatible for cloning into plasmids *pAW8-aid-Turg1:kanMX6*, *pAW8-aid-3HA-Turg1:kanMX6*, *pAW8-aid-5Myc-Turg1:kanMX6* and *pAW8-aid-V5-Turg1:kanMX6* were synthesised and cloned into their respective *Nhe*I-restricted plasmids to create *pAW8-aid-eDSR-Turg1:kanMX6*, *pAW8-aid-3HA-eDSR-Turg1:kanMX6*, *pAW8-aid-5Myc-eDSR-Turg1:kanMX6* and *pAW8-aid-V5-eDSR-Turg1:kanMX6*.

All plasmid constructs were confirmed by sequencing. All plasmid constructs and sequences are available from Addgene (see Figure 1E for Addgene ID numbers).

### 2.5. Yeast Transformations

All yeast transformations were performed using a lithium acetate method [28]. For insertion of the phleomycin/bleomycin resistance cassette adjacent to the *arg3-D4* locus, a linear PCR fragment generated using primers P19 and P20 and the plasmid template pFA6a-bleMX6 [29] was used to transform strain *arg3-D4* (AW1502). After selection on YEA plates supplemented with 30 mg/mL phleomycin, transformants were confirmed by genomic PCR and sequencing. This created strain AW1655 (*bleMX6-arg3-D4*) (Figure S1). To fuse the AID-tag at the C-terminus of Mcm4 and Rad52, primers P21 and P22 (Mcm4) or primers P23 and P24 (Rad52) were used to generate linear PCR fragments from the relevant *pAW8-aid-Turg1:kanMX6* plasmid described above and used to transform strain AW279 (WT h+). Transformants were selected on YEA plates supplemented with 200 mg/mL G418 and confirmed using genomic PCR and sequencing.

### 2.6. Spot Tests

Cell cultures grown in YE media 30 °C in YE medium were adjusted to a density of 1 × 10^7^ cells/mL. A series of 10-fold dilutions in sterile water was performed for each strain and 7 μL drops were spotted onto YEA medium plates with or without supplements.

### 2.7. Preparation of Total Cell Extract and Western Blot Analysis

Preparation of cell extracts for SDS-PAGE and Western blotting was performed as previously described [30] except NaN_3_ was added to the cell culture sample to a final concentration of 0.1% prior to centrifugation. For protein detection, the following commercially available antibodies were used. Primary antibodies: anti-V5 (Bio-Rad, # MCA1360) (diluted 1:5,000), anti-OsTIR1 (MBL, #PD048) (diluted 1:1,000) and anti-α tubulin (Sigma, #T5168) (diluted 1:10,000). Secondary antibodies: anti-mouse IgG HRP (Dako, #P0260) and anti-rabbit IgG HRP (Dako, #P0217), both diluted 1:5,000. All antibodies were incubated in phosphate-buffered saline, 0.1% *v/v* Tween 20 detergent (PBST) containing 3% skimmed milk powder except anti-OsTIR1, which was incubated in PBST containing 5% skimmed milk powder. For quantification of protein levels, Amersham ImageQuant LAS4000 Western blot imaging system was used and resulting images analysed using ImageQuant TL software (version 1.2) ( GE Healthcare, Chicago, IL, USA).

### 2.8. Synthesis of 2-[5-(adamantan-1-yl)-1H-indol-3-yl]Acetic Acid (5′-adamantyl-IAA)

5′Adamantyl-IAA was synthesised essentially as described previously [18]. Solvents were used as received, including deuterated solvents for NMR spectroscopy. NMR spectra were recorded on a Varian NMR 400 (^1^H 399.5 MHz; ^13^C{^1^H} 100.5 MHz or 500 (^1^H 499.9 MHz; 13C{^1^H} 125.7 MHz). Chemical shifts are reported in ppm. Spectra are referenced to the corresponding protic solvent (^1^H) or signals of the solvent (^13^C). Reactions were monitored by TLC on commercially available glass silica gel plates (60 Å, F254). The mobile phase was usually a solvent mixture and the visualisation was undertaken using UV light. Chromatographic purifications were carried out on an ISCO Combi Flash RF 75 or a 150 PSI purification unit. Gel columns. LC–MS purity analyses were undertaken using a 5 μm C18 110 Å column. Percentage purities were performed using a 30 min method in water/acetonitrile with 0.1% formic acid (5 min at 5%, 5−95% over 20 min, 5 min at 95%) with the UV set to 254 nm. High-resolution mass spectrometry was carried out at the University of Sussex.

#### 2.8.1. N-(4-(adamantan-1-yl)phenyl)acetamide


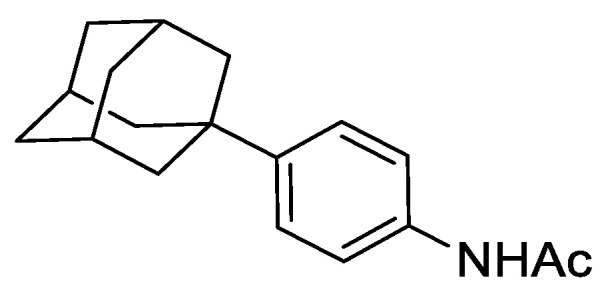


To a mixture of acetanilide (5.07 g, 37.50 mmol), 1-bromoadamantane (8.87 g, 41.25 mmol) and 1,1,2,2-tetrachloroethane (50 mL) was added zinc chloride (10.22 g, 75.00 mmol) and the resulting mixture heated to 80 °C and stirred for 72 h under an argon atmosphere. The reaction was cooled to ambient temperature and to the mixture was added 1 M aqueous HCl (150 mL) and dichloromethane (DCM) (100 mL). The resulting biphasic mixture was separated, and the aqueous phase extracted with DCM (2 × 100 mL). The combined organic extracts were dried over anhydrous MgSO_4_, filtered, and concentrated under reduced pressure. The resulting residue was purified by automated column chromatography (*n*-hexane/EtOAc, 100:0–30:70, 100 g SiO_2_). The appropriate fractions were combined and concentrated under reduced pressure to give *N*-(4-(adamantan-1-yl)phenyl)acetamide as a white solid (5.14 g, 51%). LCMS (UV, ESI) *R_t_* = 23.52 min, [M-H]^+^ *m/z* = 270.1, 88% purity. ^1^H NMR (600 MHz, *d_6_*-DMSO): *δ* = 9.82 (1H, s), 7.50–7.46 (2H, m), 7.27–7.23 (2H, m), 2.06–2.02 (3H, m), 2.01 (3H, s), 1.84–1.80 (6H, m), 1.76–1.68 (6H, m).

#### 2.8.2. N-(4-(adamantan-1-yl)-2-iodophenyl)acetamide


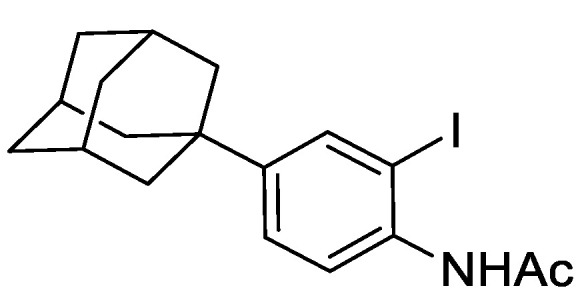


To a mixture of *N*-(4-(adamantan-1-yl)phenyl)acetamide (1.82 g, 6.76 mmol), TsOH.H_2_O (128 mg, 0.66 mmol), and MeCN (100 mL) was added NIS (1.67 g, 7.43 mmol) and the resulting mixture was stirred under an argon atmosphere for 24 h at ambient temperature. The mixture was concentrated under reduced pressure and to the residue EtOAc (50 mL) was added and saturated aqueous NaHCO_3_ (50 mL). The resulting biphasic mixture was separated, and the aqueous phase extracted with EtOAc (3 × 50 mL). The combined organic extracts were washed with brine, dried over anhydrous MgSO_4_, filtered, and concentrated under reduced pressure to give a brown solid (2.7 g). The solid was purified by automated flash column chromatography (*n*-hexane/EtOAc, 100:0–50:50, 30 g SiO_2_). The appropriate fractions were combined and concentrated under reduced pressure to give *N*-(4-(adamantan-1-yl)-2-iodophenyl)acetamide as a light orange solid (2.62 g, 98%). LCMS (UV, ESI) *R_t_* = 25.59 min, [M-H]^+^ *m/z* = 396.0, 93% purity. ^1^H NMR (600 MHz, *d_6_*-DMSO): *δ* = 9.36 (1H, s), 7.75 (1H, d, *J* = 2.2 Hz), 7.36 (1H, dd, *J* = 8.4, 2.2 Hz), 7.30 (1H, d, *J* = 8.4 Hz), 2.07–2.02 (3H, m), 2.03 (3H, s), 1.85–1.82 (6H, m), 1.76–1.69 (6H, m).

#### 2.8.3. N-(4-(adamantan-1-yl)-2-((trimethylsilyl)ethynyl)phenyl)acetamide


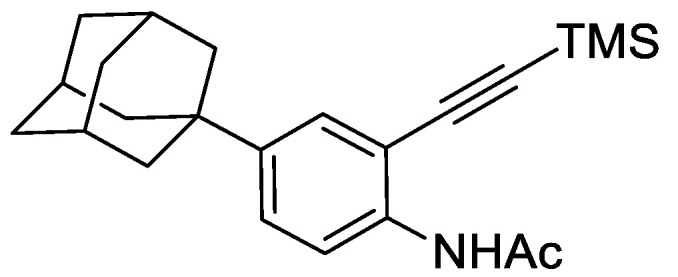


To *N*-(4-(adamantan-1-yl)-2-iodophenyl)acetamide (1.00 g, 2.53 mmol), Pd(PPh_3_)_2_Cl_2_ (44 mg, 0.063 mmol), and CuI (24 mg, 0.127 mmol) was added THF:NEt_3_ (1:1) (25 mL) followed by trimethylsilyacetylene (456 µL, 3.29 mmol). The resulting mixture was stirred under argon for 16 h at ambient temperature. The reaction was concentrated under reduced pressure and purified by automated flash column chromatography (*n*-hexane/EtOAc, 100:0–60:40, 40 g SiO_2_). The appropriate fractions were combined and concentrated under reduced pressure to give *N*-(4-(adamantan-1-yl)-2-((trimethylsilyl)ethynyl)phenyl)acetamide as a brown solid (740 mg, 80%). LCMS (UV, ESI) *R_t_* = 29.64 min, [M-H]^+^ *m/z* = did not ionise, 94% purity. ^1^H NMR (600 MHz, *d_6_*-DMSO): *δ* = 9.13 (1H, s), 7.60 (1H, d, *J* = 8.8 Hz), 7.39–7.34 (2H, m), 2.08–2.03 (6H, m), 1.85–1.81 (6H, m), 1.74–1.70 (6H, m), 0.25 (9H, s).

#### 2.8.4. 5-(Adamantan-1-yl)-1H-indole


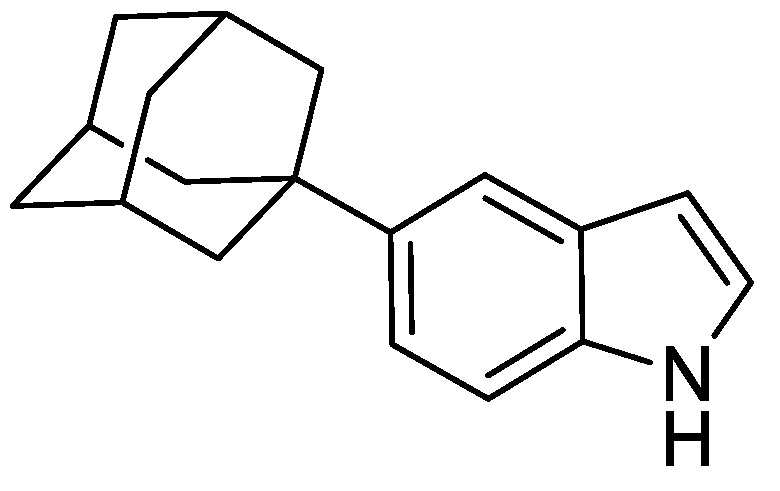


To a mixture of *N*-(4-(adamantan-1-yl)-2-((trimethylsilyl)ethynyl)phenyl)acetamide (630 mg, 1.72 mmol) and anhydrous THF (16 mL) was added tetrabutylammonium fluoride (TBAF) (1 M in THF) (2.50 mL). The resulting mixture was heated to 80 °C and stirred for 16 h under an argon atmosphere. The reaction was cooled to ambient temperature and to the mixture was added EtOAc (15 mL) and water (15 mL). The resulting biphasic mixture was separated, and the aqueous phase extracted with EtOAc (3 × 15 mL). The combined organic extracts were washed with brine (25 mL), dried over anhydrous MgSO_4_, filtered, and concentrated under reduced pressure to give a brown oil (500 mg). The resulting residue was purified by automated flash column chromatography (*n*-hexane/EtOAc, 100:0–70:30, 24 g SiO_2_). The appropriate fractions were combined and concentrated under reduced pressure to give 5-(adamantan-1-yl)-1*H*-indole as a pale yellow solid (330 mg, 76%). LCMS (UV, ESI) *R_t_* = 27.19 min, [M-H]^+^ *m/z* = 252.1, 91% purity. ^1^H NMR (600 MHz, *d_6_*-DMSO): *δ* = 10.88 (1H, s), 7.44 (1H, s), 7.30 (1H, d, *J* = 8.8 Hz), 7.26 (1H, m), 7.14 (1H, dd, *J* = 8.8, 1.8 Hz), 6.35 (1H, m), 2.09–2.05 (3H, m), 1.92–1.89 (6H, m), 1.78–1.72 (6H, m).

#### 2.8.5. Methyl 2-(5-(adamantan-1-yl)-1H-indol-3-yl)-2-oxoacetate


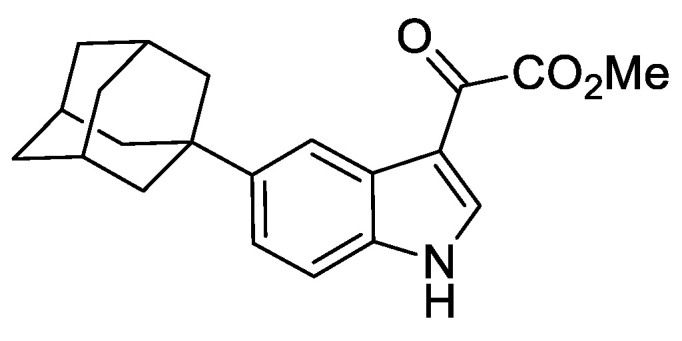


To a mixture of 5-(adamantan-1-yl)-1*H*-indole (294 mg, 1.17 mmol) and anhydrous Et_2_O (10 mL) was added oxalyl chloride (148 µL, 1.75 mmol) and the resulting mixture stirred for 16 h at ambient temperature under argon. To the mixture was added MeOH (10 mL) and the resulting mixture stirred for 15 min. The resulting mixture was concentrated under reduced pressure to give a yellow solid of sufficient purity to be utilised in the subsequent reaction without further manipulation.

#### 2.8.6. 2-[5-(Adamantan-1-yl)-1H-indol-3-yl]Acetic Acid


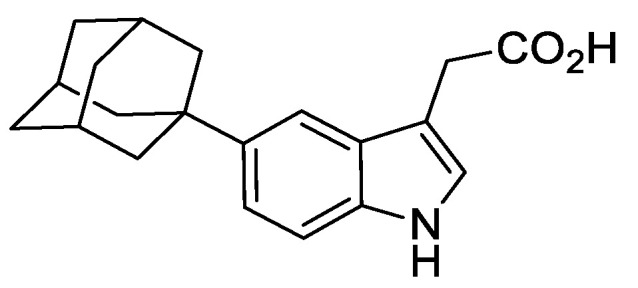


To a mixture of crude methyl 2-(5-(adamantan-1-yl)-1*H*-indol-3-yl)-2-oxoacetate (395 mg, 1.17 mmol), NaH_2_PO_2_.H_2_O (1.24 g, 11.70 mmol), and H_2_O/1,4-dioxane (1:5) (10 mL) was added Pd/C (10%) (128 mg, 0.12 mmol). The resulting mixture was heated to 100 °C and stirred for 72 h under an argon atmosphere. The reaction was cooled to ambient temperature and filtered through Celite^®^, washing with EtOAc (25 mL). To the mixture was added 2M aqueous HCl (25 mL) and the resulting biphasic mixture separated. The organic phase was washed with brine (25 mL), dried over anhydrous MgSO_4_, filtered, and concentrated under reduced pressure to give a light red gum (550 mg). The resulting residue was purified by automated flash column chromatography (*n*-hexane/EtOAc, 100:0–70:30, 24 g SiO_2_). The appropriate fractions were combined and concentrated under reduced pressure to give 2-(5-(adamantan-1-yl)-1*H*-indol-3-yl)acetic acid as a white solid (129 mg, 36% over 2 steps). LCMS (UV, ESI) *R_t_* = 22.49 min, [M-H]^+^ *m/z* = 310.0, 96% purity. ^1^H NMR (600 MHz, *d_6_*-DMSO): *δ* = 12.11 (1H, s), 10.73 (1H, s), 7.42 (1H, m), 7.27 (1H, d, *J* = 8.8 Hz), 7.18–7.13 (2H, m), 3.62 (2H, s), 2.10–2.05 (3H, m), 1.93–1.89 (6H, m), 1.78–1.72 (6H, m). ^13^C NMR (151 MHz, *d_6_ −*DMSO): *δ* = 173.2, 141.1, 134.3, 126.9, 123.8, 118.5, 113.7, 110.8, 107.7, 43.4, 36.4, 35.5, 31.0, 28.5. HRMS (ESI-[+Na]) *m/z*: Calcd for C_20_H_23_NNaO_2_ 332.1626; Found 332.1615.

## 3. Results

### 3.1. Integration of OsTIR1^WT^ and OsTIR1^F74A^ Expression Constructs at the arg3-D4 Locus

OsTIR1 is the most commonly used AID system auxin receptor F-box protein. Moreover, in budding yeast, OsTIR1 has been shown to work at high temperatures and expression of OsTIR1 from the constitutive alcohol dehydrogenase gene (*ADH1*) promoter does not affect cell growth [1]. We therefore wished to test OsTIR1 not fused to Skp1 in a new fission yeast AID system.

We developed a set of plasmids to integrate the OsTIR1^WT^ and OsTIR1^F74A^ expression constructs at the *S. pombe arg3-D4* locus. We sequenced the *arg3-D4* locus [24] (Figure S1) to determine the region deleted and synthesised this sequence and approximately 500bp flanking the deletion site. These flanking sequences allow integration of the deleted *arg3* gene fragment at the *arg3-D4* locus and transformants can be screened for arginine prototrophy. Synthesis of this fragment allowed for insertion of an MCS (*Nhe*I-*Nde*I-*Sph*I-*Xma*I-*Sac*I-*Pst*I-*Sal*I-*Spe*I) and the removal of restriction enzyme recognition sites from the *arg3* gene sequence which may hinder subsequent cloning events. The synthesised gene fragment was cloned into pUC19* (where * denotes the removal of the *Nde*I restriction enzyme recognition site from the plasmid backbone) to create MCS-*Sp.arg3+*/pUC19*.

Expression cassettes consisting of an MCS site flanked by approximately 820bp of the *S. pombe adh1* promoter and 160bp of the *S. cerevisiae ADH1* terminator sequences were constructed. Three individual constructs were created each containing different *adh1* promoter strengths. The strongest promoter strength was the wild-type *adh1* promoter sequence (*P_adh1_*) followed by *adh15* promoter (*P_adh15_*), a weak derivative of the *adh1* promoter [11] and finally the *adh81* promoter (*P_adh81_*) a much weaker derivative of *P_adh1_* [31]. These three expression constructs were cloned into MCS-*Sp.arg3+*/pUC19* to create P_adh1_-MCS-T_ADH1_-*Sp.arg3+*/pUC19*, P_adh15_-MCS-T_ADH1_-*Sp.arg3+*/pUC19* and P_adh81_-MCS-T_ADH1_-*Sp.arg3+*/pUC19*, respectively (MCS = *Nde*I-*Xma*I-*Sac*I-*Pst*I-*Sal*I).

For the new AID system, human-codon optimised OsTIR1^WT^-NLS (NLS: nuclear localisation signal) was gene synthesised and cloned into P_adh1_-MCS-T_ADH1_-*Sp.arg3+/*pUC19* to create P_adh1_-OsTIR1^WT^-NLS-T_ADH1_-*Sp.arg3+*/pUC19*. For AID version 2 (AID2), human-codon optimised OsTIR1^F74A^-NLS was gene synthesised and cloned into P_adh1_-MCS-T_ADH1_-*Sp.arg3+/*pUC19* to create P_adh1_-OsTIR1^F74A^-NLS-T_ADH1_-*Sp.arg3+*/pUC19*. The OsTIR1^F74A^ mutation corresponds to the AtTIR1 auxin-binding site mutation F79A identified by Yamada et al. [18] and used by Nishimura et al. [19] (see also Figure S3). The NLS directs the system to specifically deplete nuclear proteins. The plasmids P_adh1_-OsTIR1^WT^-NLS-T_ADH1_-arg3+/pUC19* and P_adh1_-OsTIR1^F74A^-NLS-T_ADH1_-*Sp.arg3+*/pUC19* were restricted with *Not*I restriction enzyme and the linear fragments used to transform AW1655 (*bleMX6-arg3-D4*). Transformants were screened for arginine prototrophy and successful integrants confirmed by genomic PCR and sequencing (Figure 1B). This created strains *Padh1-OsTIR1^WT^* (AW1762) and *Padh1-OsTIR1^F74A^* (AW1703). Expression of OsTIR1^WT^ and OsTIR1^F74A^ was confirmed by immunoblot analysis and, as shown previously [17], the F74A substitution does not significantly affect stability of the TIR1 protein (Figure 1C). Expression of OsTIR1^WT^ or OsTIR1^F74A^ from the strong constitutive *adh1* promoter does not affect cell growth rate, with doubling times of *Padh1-OsTIR1^WT^* and *Padh1-OsTIR1^F74A^* cells comparable to wild-type cells (Figure 1D). As cellular levels of TIR1 have been shown to be a limiting factor for auxin response [10,32,33], we predict that increasing the TIR1 expression level will increase AID efficiency.

### 3.2. Plasmids for C-Terminal AID Tagging

To allow the C-terminal tagging of proteins of interest, we developed a set of plasmids containing the full-length *A. thaliana* auxin-responsive AUX/IAA protein IAA17 gene sequence. The tagging constructs also include a sequence encoding a flexible 10-amino-acid linker (Gly-Gly-Ser-Gly-Gly-Ser-Gly-Ser-Gly-Ala) that will be inserted between the protein of interest and the AID-tag. This was included to help minimise loss of either target protein or AID-tag function. The plasmids also included approximately 530 bp of *S. pombe urg1* downstream sequence to act as a transcription terminator and the antibiotic resistance cassette, kanMX6, which allows selection of G418-resistant cells (Figure 1E).

To detect AID-tagged proteins by immunoblotting using commercial antibodies, a series of additional short epitope tag sequences were fused to the C-terminus of the AID tag. These tags included three copies of the influenza virus hemagglutinin (HA) epitope tag (3HA), five copies of the human c-myc (Myc) epitope (5Myc) and a single copy of the V5 tag derived from the P and V protein of the simian virus 5 (SV5, a paramyxovirus). The AID and epitope tags were separated by a short 6-amino-acid flexible linker (Gly-Ala-Gly-Ala-Gly-Ala) (Figure 1E).

To provide an option to constitutively reduce AID-tagged protein expression levels from the constructs outlined above, we inserted the 157 bp DSR element derived from the *S. pombe spo5* gene directly downstream of the tag stop codon (Figure 1E). Levels of yEGFP expressed from the uracil-inducible *urg1* locus are reduced approximately 25-fold by the *spo5DSR* element compared to the no DSR control [23]. The *spo5*DSR element contains 5 copies of the hexanucleotide motif U(U/C)AAAC shown to be highly enriched in DSR elements [22]. To increase the efficiency of the *spo5*DSR element and further reduce AID-tagged protein levels, two sequences similar to the core motif were mutated to match the hexanucleotide DSR motif (Figure S2). This new DSR element, termed ‘eDSR’, was introduced into the AID-plasmids, replacing *spo5*DSR. The full set of plasmids are shown in Figure 1E.

The AID-tagging constructs were cloned into the Cre-expression plasmid pAW8, allowing for rapid and efficient C-terminal fusion of the AID-tag using Cre-mediated cassette exchange [27]. These tagging construct plasmids are also compatible with PCR-based genomic strategies with the primer sequences required to amplify the series of AID constructs shown in Figure 1F.

### 3.3. Testing the New S. pombe AID/OsTIR1(WT) System

As a target of degradation, we used Mcm4, a component of the DNA replication MCM complex to test the new AID/OsTIR1 system. Mcm4 is an essential protein and *mcm4*Δ cells are inviable. The *i*-AID system (*Padh15-Skp1-AtTIR1*) was used previously to deplete Mcm4 [10] and allows direct comparison of the efficiency of the two systems. Mcm4 was successfully C-terminally AID-tagged using a PCR-mediated approach using the plasmids outlined in Figure 1E. The *mcm4-AID* strain was then crossed to strains expressing *Padh15-skp1-AtTIR1* or *Padh1-OsTIR1^WT^* and the resulting strains were spotted onto a YEA plate or YEA plates containing the synthetic auxin NAA at 500 μM and the plates were incubated at 25 °C, 30 °C or 36 °C for 3 days (Figure 1G). Unlike Skp1-AtTIR1 expressing *i*-AID cells, expression of OsTIR1 from the strong *adh1* constitutive promoter showed no inhibition in cell growth, as has previously been observed for budding yeast [1]. Although *mcm4-AID skp1-AtTIR1* cell growth was severely inhibited at 25 °C by the presence of auxin, increasing the temperature to 30 °C reduced this inhibition and growth rates were more similar to WT (Figure 1G). However, *mcm4-aid OsTIR1 ^WT^* cells showed complete loss of cell viability when grown in the presence of auxin at 25 °C and, more importantly, also at 30 °C (Figure 1G). The relative thermostability of OsTIR1 compared to AtTIR1 has been previously reported [1] and explains the improved performance observed for our new AID system with respect to temperature. Overall, these data clearly demonstrate the significant improvement in degradation efficiency when using *Padh1-OsTIR1* as opposed to systems involving *Padh15-skp1-AtTIR1*.

### 3.4. Further Characterisation of the S. pombe AID/OsTIR1^WT^ System

**Figure 2:**
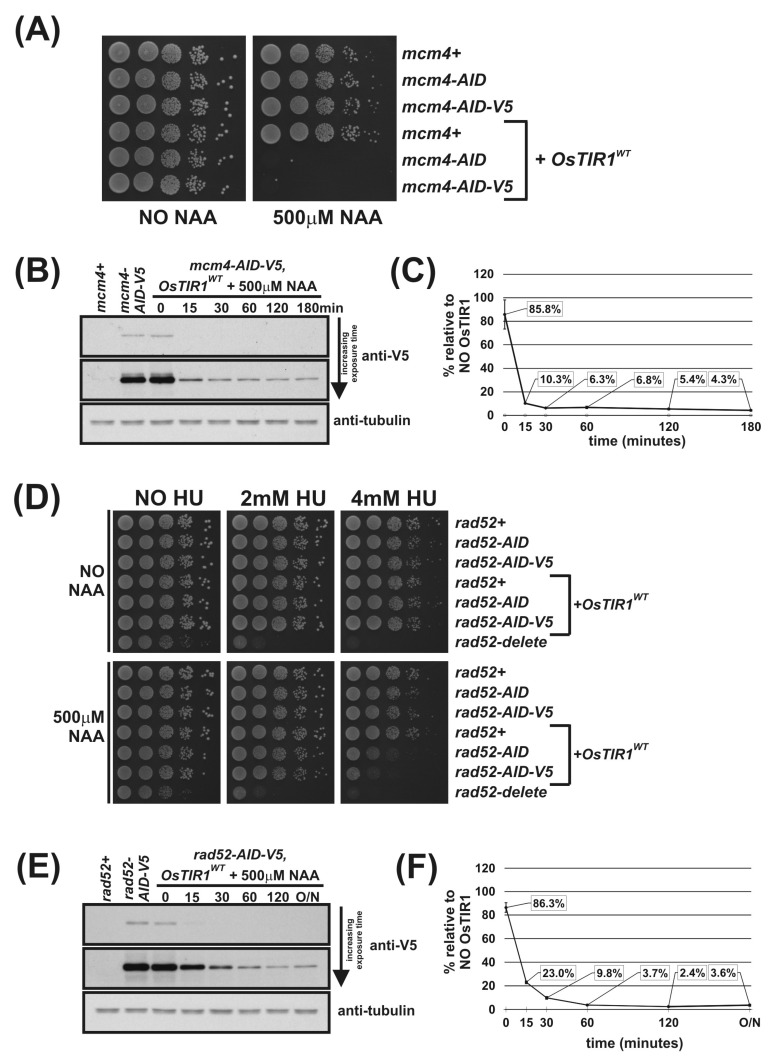
Regulation of Mcm4 and Rad52 activity using AID/OsTIR1^WT^. (**A**) Isogenic strains with indicated genotypes (*mcm4+* (AW279), *mcm4-AID* (AW1893), *mcm4-AID-V5* (AW1899), *OsTIR1^WT^* (AW1762), *mcm4-AID OsTIR1^WT^* (AW1923), *mcm4-AID-V5 OsTIR1^WT^* (AW1935)) were spotted onto YEA plates and plates containing 500 μM NAA. Plates were grown at 30 °C for 3 days. The experiment was repeated and similar results were obtained. (**B**) Representative immunoblot on total protein extract from *mcm4+* (AW279), *mcm4-AID-V5* (AW1899) and *mcm4-AID-V5 OsTIR1^WT^* (AW1935) cells treated with 500 μM NAA and sampled at indicated time points and detected using anti-V5 antibody. Anti-tubulin was used as a loading control. (**C**) Graph of quantified anti-V5 signal normalised against anti-tubulin signal for (**B**) (data represented as the mean values +/− SD of three independent experiments). (**D**) Isogenic strains with indicated genotypes (*rad52+* (AW279), *rad52-AID* (AW1901), *rad52-AID-V5* (AW1907), *OsTIR1^WT^* (AW1762), *rad52-AID OsTIR1^WT^* (AW1990), *rad52-AID-V5 OsTIR1^WT^* (AW1996), *rad52*Δ (AW1581)) were spotted onto YEA plates and plates containing 500 μM NAA and/or genotoxic agent hydroxyurea (HU) at indicated concentrations. Plates were grown at 30 °C for 3 days. The experiment was repeated and similar results were obtained. (**E**) Representative immunoblot on total protein extract from *rad52+* (AW279), *rad52-AID-V5* (AW1907) and *rad52-AID-V5 OsTIR1^WT^* (AW1996) cells treated with 500 μM NAA and sampled at the indicated time points and detected using anti-V5 antibody. Anti-tubulin was used as a loading control. (**F**) Graph of quantified anti-V5 signal normalised against anti-tubulin signal (data represented as the mean values +/− SD of three independent experiments).

To determine the rate and percentage reduction in AID-tagged protein levels following addition of auxin, we created the strains *mcm4-AID-5myc* and *mcm4-AID-V5* using the plasmids outlined in Figure 1E (we were unable to create *mcm4-AID-3HA*). As we found the signal-to-noise ratio of the anti-V5 antibody was significantly higher than that for anti-Myc, *mcm4-AID-V5* cells were used to study protein levels. Complete inviability in the presence of NAA of *mcm4-AID-V5 OsTIR1^WT^* cells demonstrate that the V5 epitope tag does not compromise AID degron function (Figure 2A).

*mcm4-AID-V5 OsTIR1^WT^* cells were grown to mid-log phase, synthetic auxin NAA was added to a final concentration of 500 μM and total protein extracted at various timepoints. Total protein was also extracted from *mcm4*^+^ (untagged Mcm4) and *mcm4-AID-V5* cells with no OsTIR1. Following immunoblotting using anti-V5 antibodies and, as a loading control, anti-tubulin, the degradation kinetics were determined (Figure 2B). Quantification of AID-tagged protein levels reveal a 14% reduction in Mcm4-AID-V5 protein levels in cells exogenously expressing OsTIR1 in the absence of NAA. The chronic auxin-independent depletion of AID-tagged proteins by TIR1 has been extensively reported [34,35,36]. After addition of NAA a rapid depletion of cellular levels of Mcm4-AID-V5 was observed with a 90% reduction after 15 min. However, detectable levels of Mcm4-AID-V5 were observed 3 h after addition of NAA with approximately 5% of protein remaining (Figure 2C).

Using Mcm4 to assess the efficiency of the OsTIR1 auxin-inducible degron system is limited because Mcm4 is an essential protein and a reduction in protein level by approximately 95% leads to complete cell inviability (Figure 2A,C). Therefore, we chose to also study the DNA repair protein Rad52 because, unlike Mcm4, Rad52 is a non-essential protein and *rad52*Δ cells are viable. Depletion of Rad52 can be monitored phenotypically as *rad52*Δ cells display a slow-growth phenotype and are hyper sensitive to DNA damaging agents such as hydroxyurea (HU) [37].

We successfully AID-tagged Rad52 via a PCR-based approach using the plasmids outlined in Figure 1E as PCR templates. Spot test analysis shows that *rad52-AID* and *rad52-AID-V5* cells grow at WT rates and show WT levels of resistance to the genotoxic agent HU, meaning that the AID and AID-V5 tags do not adversely affect Rad52 function (Figure 2D). This is in contrast to *rad52*Δ cells that are both slow growing and are hypersensitive to HU treatment with significant levels of cell death observed at low HU concentrations (2 mM). In the presence of NAA, however, *rad52-AID OsTIR1^WT^* and *rad52-AID-V5 OsTIR1^WT^* cells exhibit increased sensitivity to HU when compared to untagged Rad52 control cells, but only at higher HU concentrations (4 mM). This indicates that significant levels of Rad52-AID protein remain when NAA is present (Figure 2D).

We used *rad52-AID-V5* cells to monitor protein levels. *rad52-AID-V5 OsTIR1^WT^* cells were grown to mid-log phase, NAA was added to a final concentration of 500 μM and total protein extracted at various timepoints. As with *mcm4-AID-V5 OsTIR1^WT^* cells, chronic auxin-independent depletion of Rad52-AID-V5 by OsTIR1 was observed with protein levels reduced by approximately 14% compared to r*ad52-AID-V5* cells (Figure 2F). As with Mcm4, the addition of NAA induced a rapid depletion of Rad52-AID-V5, with levels reduced by 78% within 15 min but Rad52-AID-V5 protein levels were detectable even after overnight treatment (18 h). Residual levels of Rad52-AID-V5 in cells expressing OsTIR1 in the presence of NAA are comparable to those seen for Mcm4—approximately 4% relative to no TIR1 (Figure 2F).

Overall, these data demonstrate the effectiveness of the AID/OsTIR1^WT^ system. AID-tagged proteins are rapidly degraded in the presence of NAA with overall levels reduced approximately 20-fold. However, detectable levels of AID-tagged protein remain. For an essential protein such as Mcm4, this residuary level is low enough to render the cell inviable and thus a conditional null-mutant phenotype was obtained. However, for a non-essential gene, such as Rad52, this residual level did not manifest a conditional null phenotype: NAA-treated cells only showed sensitivity to HU at concentrations that were higher than those required to kill *rad52*-deleted cells. Moreover, the effectiveness of the AID/OsTIR1^WT^ system is potentially compromised by the NAA-independent association of AID-tagged proteins with OsTIR1, resulting in levels being reduced by approximately 14% relative to no TIR1 controls.

### 3.5. Using the AID2/OsTIR1^F74A^ System in S. pombe

Recent AID systems have made use of synthetic auxin–TIR1 pairs that function orthogonally to the natural auxin signalling pathway. The systems involve an auxin-binding site mutant and a 5-substituted synthetic auxin and overcome problems associated with the original AID system, namely, the requirement for a high dose of auxin and the auxin-independent degradation of AID-tagged target proteins. The new AID systems (termed AID version 2 or ‘AID2′) have been characterised for use in chicken DT40 cells, budding yeast, mammalian cells and mice and have been shown to require a markedly lower ligand concentration for the association of TIR1 with the AID-tag and no detectable basal degradation of AID-tagged proteins was observed [6,19]. The rate of degradation has been also shown to be even quicker in AID2 compared to AID [6].

**Figure 3:**
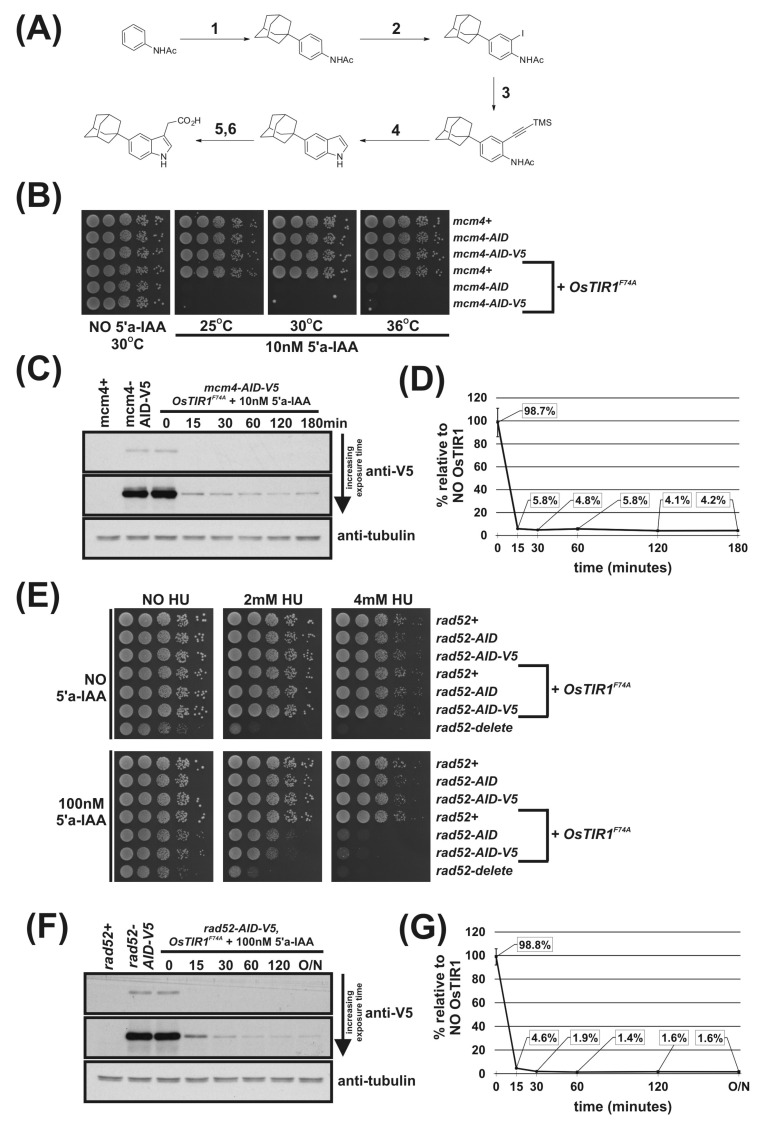
Regulation of Mcm4 and Rad52 activity using AID2/OsTIR1^F74A^. (**A**) Schematic illustration showing synthesis of 2-[5-(adamantan-1-yl)-1H-indol-3-yl]acetic acid (5′adamantyl-IAA). (1) 1-Bromoadamantane, ZnCl_2_, 1,1,2,2-tetrachloroethane, 80 °C, 72 h, 51%; (2) NIS, TsOH.H_2_O, MeCN, rt, 24 h, 98%; (3) trimethylsilyacetylene, Pd(PPh_3_)_2_Cl_2_, CuI, THF:NEt_3_ (1:1), 80%; (4) TBAF, THF, 80 °C, 16 h, 76%; (5) oxalyl chloride, Et_2_O, rt, 16 h; (6) NaH_2_PO_2_.H_2_O, Pd/C, H_2_O/1,4-dioxane (1:5), 100 °C, 72 h, 36% over two steps. (**B**) Isogenic strains with the indicated genotypes (*mcm4+* (AW279), *mcm4-AID* (AW1893), *mcm4-AID-V5* (AW1899), *OsTIR1^F74A^* (AW1703), *mcm4-AID OsTIR1^F74A^* (AW1925), *mcm4-AID-V5 OsTIR1^F74A^* (AW1937)) were spotted onto YEA plates and plates containing 10 nM 5′adamantyl-IAA (5′a-IAA). Plates were grown at indicated temperatures for 3 days. The experiment was repeated and similar results were obtained. (**C**) Representative immunoblot on total protein extract from *mcm4+* (AW279), *mcm4-AID-V5* (AW1899) and *mcm4-AID-V5 OsTIR1^F74A^* (AW1937) cells treated with 10 nM 5′adamantyl-IAA (5′a-IAA) and sampled at the indicated time points and detected using anti-V5 antibody. Anti-tubulin was used as a loading control. (**D**) Graph of quantified anti-V5 signal normalised against anti-tubulin signal for (C) (data represented as the mean values +/− SD of three independent experiments). (**E**) Isogenic strains with the indicated genotypes (*rad52+* (AW279), *rad52-AID* (AW1901), *rad52-AID-V5* (AW1907) *OsTIR1^F74A^* (AW1703), *rad52-AID OsTIR1^F74A^* (AW1962), *rad52-AID-V5 OsTIR1^F74A^* (AW1968), *rad52*Δ (AW1581)) were spotted onto YEA plates and plates containing 100 nM 5′adamantyl-IAA (5′a-IAA) and/or genotoxic agent hydroxyurea (HU) at the indicated concentrations. Plates were grown at 30 °C for 3 days. The experiment was repeated and similar results were obtained. (**F**) Representative immunoblot on total protein extract from *rad52+* (AW279), *rad52-AID-V5* (AW1907) and *rad52-AID-V5 OsTIR1^F74A^* (AW1968) cells treated with 100 nM 5′adamantyl-IAA (5′a-IAA) and sampled at indicated time points and detected using anti-V5 antibody. Anti-tubulin was used as a loading control. (**G**) Graph of quantified anti-V5 signal normalised against anti-tubulin signal for (**F**) (data represented as the mean values +/− SD of three independent experiments).

To test an AID2 in fission yeast, we construct a system analogous to that described by Yamada et al. [18]. We synthesised the 5-substituted auxin 5′adamantyl-IAA (Figure 3A) and created a strain that endogenously expresses the high-affinity-binding partner OsTIR1^F74A^, corresponding to AtTIR1^F79A^, using the *arg3-D4* integration expression vector system we have developed (Figure 1B). Growth analysis showed that overexpression of OsTIR1^F74A^ using the strong constitutive *adh1* promoter does not affect cell growth (Figure 1D). We crossed the *OsTIR1^F74A^* strain to the *mcm4-AID* and *mcm4-AID-V5* strains and, using spot test analysis, tested a range of concentrations of the AID2 inducer 5′adamantyl-IAA (100 nM, 10 nM and 1 nM). We found that a 5′adamantyl-IAA concentration of 1 nM was sufficient to generate a lethal phenotype and that 10 nM was, upon microscopic examination, able to completely inhibit cell growth (Figure S5A and Figure 3B). The 10 nM represents a 5,000-fold reduction in ligand concentration compared to OsTIR1^WT^/NAA. Interestingly, the AID2/OsTIR1^F74A^ system also functions better at the higher growth temperature of 36 °C (Figure 3B; compare with Figure 1G). This clearly demonstrates that the AID2 OsTIR1^F74A^/5′adamantyl-IAA system is fully functional in fission yeast and, with respect to Mcm4, requires a significantly lower ligand concentration compared to AID/OsTIR1^WT^/NAA.

Immunoblot analysis shows no auxin-independent depletion of Mcm4-AID-V5 in cells expressing OsTIR1^F74A^ (Figure 3C,D) in contrast to *mcm4-AID-V5 OsTIR1^WT^* cells (Figure 2B,C). Furthermore, Figure 3C,D show that the kinetics of depletion are faster in *mcm4-AID-V5 OsTIR1^F74A^* cells after the addition of 5′adamantyl-IAA (94% reduction after 15 min) when compared to *mcm4-AID-V5 OsTIR1^WT^* cells treated with NAA (see Figure 2B,C) (90% reduction after 15 min). These faster kinetics may be due to the higher affinity of the 5′adamantyl-IAA-induced OsTIR1^F74A^ interaction when compared to the NAA-induced OsTIR1^WT^ interaction [18]. Surprisingly, steady-state proteins levels after addition of 5′adamantyl-IAA to *mcm4-AID-V5 OsTIR1^F74A^* cells are similar to those seen for *mcm4-AID-V5 OsTIR*1*^WT^* cells after NAA addition (approximately 4% of no TIR1 levels).

To test AID2 using Rad52, *rad52-AID OsTIR1^F74A^* and *rad52-AID-V5 OsTIR1^F74A^* cells were created. We tested a range of 5′adamantyl-IAA concentrations (1,000 nM, 100 nM and 10 nM) using spot test analysis of *rad52-AID OsTIR1^F74A^* cells (Figure S5B) and determined that a concentration of 100 nM 5′adamantyl-IAA was required to maximally induce the *rad52* phenotype—a 500-fold lower concentration as compared to NAA. The Rad52-AID/OsTIR1^F74A^ system is also clearly more efficient than the Rad52-AID/OsTIR1^WT^ system. Growth rates of *rad52-AID OsTIR1^F74A^* cells were reduced in the presence of 5′adamantyl-IAA compared to *rad52-AID cells* (Figure 3E), whereas *rad52-AID OsTIR1^WT^* cells treated with NAA do not show reduced growth rate (Figure 2D). Furthermore, *rad52-AID OsTIR1^F74A^* cells treated with 5′adamantyl-IAA show a marked reduction in cell viability when grown in the presence of HU (Figure 3E) when compared to *rad52-AID OsTIR1^WT^* cells treated with NAA (Figure 2D). However, despite improved efficiency of Rad52 degradation by AID2, *rad52-AID/OsTIR1^F74A^* cells treated with 5′adamantyl-IAA are not as slow growing nor as sensitive to HU as *rad52*Δ cells, implying residuary Rad52 activity remains in these cells.

As seen for Mcm4-AID-V5, immunoblot analysis shows no auxin-independent depletion of Rad52-AID-V5 in cells expressing OsTIR1^F74A^ (Figure 3F,G). Furthermore, Figure 3G shows that the kinetics of depletion are faster in *rad52-AID-V5 OsTIR1^F74A^* cells after the addition of 5′adamantyl-IAA (94% reduction after 15 min) when compared to r*ad52-AID-V5 OsTIR1^WT^* cells treated with NAA (Figure 2B,C) (77% reduction after 15 min). The steady-state protein levels after addition of 5′adamantyl-IAA to *rad52-AID-V5 OsTIR1^F74A^* cells are significantly lower than those seen for *rad52-AID-V5 OsTIR1^WT^* cells treated with NAA (1.6% and 3.6%, respectively), which explains the slower growth rates and increased HU sensitivity described above.

As recently shown by others [6], the AID2 (OsTIR1^F74A^/5′adamantyl-IAA) system is clearly an improvement on the AID (OsTIR1^WT^/NAA) system with faster degradation kinetics, much lower ligand concentrations and no auxin-independent depletion of AID-tagged proteins. However, despite this increase in functionality, observable levels of AID-tagged proteins remain.

### 3.6. Use of DSR Elements Allows Generation of Stricter S. pombe Mutants

Despite significantly improving the fission yeast auxin-dependent degron system, the reduction in protein levels was insufficient to obtain a conditional *rad52*-null mutant phenotype. To achieve such a phenotype, other levels of regulation are therefore required. The regulation of transcription is an effective and well-defined option but requires additional genome editing with the insertion of a regulatable promoter sequence and the manipulation of the growth media in order to downregulate transcription. Determinant of Selective Removal (DSR) elements have previously been shown to effectively modulate protein levels [23]. As DSR elements are usually located at the 3′ end of the transcript they can easily be incorporated into the AID-plasmid sequence (Figure 1E) and therefore require no extra steps. The use of DSRs does, however, have the disadvantage that protein levels are constitutively lowered.

**Figure 4:**
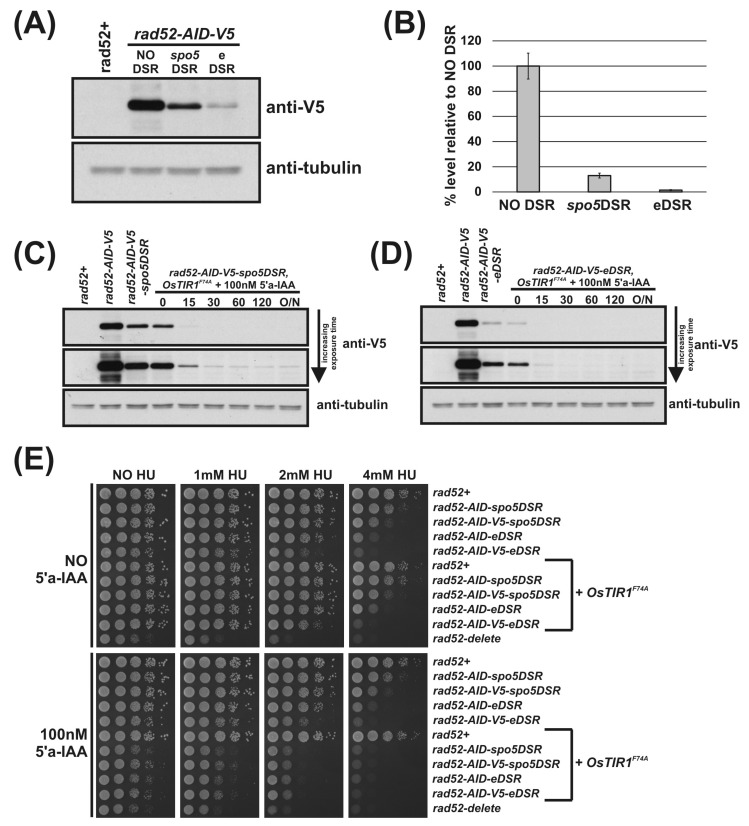
DSR elements improve AID2/OsTIR1^F74A^. (**A**) Representative immunoblot on total protein extract from *rad52+* (AW279), *rad52-AID-V5* (NO DSR) (AW1907), *rad52-AID-V5-spo5DSR* (*spo5*DSR) (AW1921) and *rad52-AID-V5-eDSR* (eDSR) (AW2017) cells detected using anti-V5 antibody. Anti-tubulin was used as a loading control. (**B**) Histogram of quantified anti-V5 signal normalised against anti-tubulin signal for (**A**) (data represented as the mean values +/− SD of three independent experiments). (**C**) Representative immunoblot on total protein extract from control and *rad52-AID-V5-spo5DSR OsTIR1^F74A^* (AW1976) cells treated with 100 nM 5′adamantyl-IAA (5′a-IAA) and sampled at indicated time points and detected using anti-V5 antibody. Anti-tubulin was used as a loading control. (**D**) Representative immunoblot on total protein extract from control and *rad52-AID-V5-eDSR OsTIR1^F74A^* (AW2026) cells treated with 100 nM 5′adamantyl-IAA (5′a-IAA) and sampled at the indicated time points and detected using anti-V5 antibody. Anti-tubulin was used as a loading control. (**E**) Isogenic strains with indicated genotypes (*rad52+* (AW279), *rad52-AID-spo5DSR* (AW1915), *rad52-AID-V5-spo5DSR* (AW1921), *rad52-AID-eDSR* (AW2011), *rad52-AID-V5-eDSR* (AW2017), *OsTIR1^F74A^* (AW1703), *rad52-AID-spo5DSR*) *OsTIR1^F74A^* (AW1970), *rad52-AID-V5-spo5DSR OsTIR1^F74A^* (AW1976), *rad52-AID-eDSR OsTIR1^F74A^* (AW2024), *rad52-AID-V5-eDSR OsTIR1^F74A^* (AW2026), *rad52*Δ (AW1581)) were spotted onto YEA plates and plates containing 100 nM 5′adamantyl-IAA (5′a-IAA) and/or genotoxic agent hydroxyurea (HU) at the indicated concentrations. Plates were grown at 30 °C for 3 days. Experiment was repeated and similar results obtained.

Using the DSR containing AID-plasmids outlined in Figure 1E, we C-terminally tagged the *rad52* locus with AID-*spo5*DSR, AID-eDSR, AID-V5-*spo5*DSR and AID-V5-eDSR. To determine the relative effectiveness of the *spo5*DSR and eDSR on Rad52-AID-V5 levels, Western blot analysis was performed. Immunoblot of total protein extracted from logarithmically growing *rad52-AID* (NO DSR), *rad52-AID-spo5DSR* and *rad52-AID-eDSR* cells shows the effect of DSR elements on constitutively reducing protein levels (Figure 4A). The previously untested eDSR element—a mutant form of *spo5*DSR engineered to contain two additional copies of the DSRcore element—is more effective at reducing levels than *spo5*DSR. Quantification of Rad52-AID-V5 proteins levels revealed that the *spo5*DSR element reduces the level on average by 84% and the eDSR element by 98% (Figure 4B). As above, addition of 5′adamantyl-IAA to *rad52-AID-spo5DSR* and *rad52-AID-eDS*R cells exogenously expressing OsTIR1^F74A^ caused rapid depletion of Rad52-AID-tagged proteins (Figure 4C,D, respectively). Interestingly, in *rad52-AID-V5-spo5DSR* cells treated with 5′adamantyl-IAA, steady-state protein levels were still detectable despite a reduction in initial protein levels by 84% (Figure 4B,C). This suggests that as the AID-tagged protein level is reduced, the efficiency of degradation is also reduced. However, in *rad52-AID-V5-eDSR* cells treated with 5′adamantyl-IAA, where initial levels are reduced by 98%, steady-state protein levels were un-detectable (Figure 4D).

The effects of the reduction in Rad52-AID protein levels due to the DSR elements are revealed by spot test analysis (Figure 4E). Predictably, as levels of Rad52 decrease in *rad52-AID* (NO DSR), *rad52-AID-spo5DSR* and *rad52-AID-eDSR* cells, sensitivity to 4 mM HU increases. Interestingly, HU sensitivity was slightly increased when a V5 tag for each of these strains was also present, implying that the V5 tag reduces Rad52 activity (Figure 4E). In the presence of 100 nM 5′adamantyl-IAA, *rad52-AID-spo5DSR OsTIR1^F74A^* cells exhibit a slow growth phenotype that is significantly more pronounced as seen for *rad52-AID/OsTIR1^F74A^* cells, but not as slow growing as *rad52*Δ cells (Figure 4E). This pattern is repeated for plates supplemented with HU. *rad52-AID-spo5DSR OsTIR1^F74A^* cells exhibit sensitivity to HU that is significantly more severe than that seen for *rad52-AID OsTIR1^F74A^* cells but not as sensitive as *rad52*Δ cells. Unexpectedly, when grown in the presence of 5′adamantyl-IAA, *rad52-AID-eDSR OsTIR1^F74A^* cells are as slow growing and as sensitive to HU as *rad52-AID-spo5DSR OsTIR1^F74A^* cells. This was surprising as the level of Rad52-AID-V5 protein is clearly lower when associated with eDSR than that of Rad52-AID-V5 associated with *spo5*DSR (Figure 4C,D, respectively). This may reflect the sensitivity limit of the spot test assay.

The use of DSR elements clearly reduces AID-target protein levels leading to a more penetrative phenotype. Frustratingly, despite the higher efficiency of the eDSR element compared to *spo5*DSR, *rad52-AID-eDSR OsTIR1^F74A^* and *rad52-AID-spo5DSR OsTIR1^F74A^* are as equally sensitive to HU when 5′adamantyl-IAA is present (Figure 4E) and neither are as sensitive as *rad52*Δ cells. However, the residual level of Rad52 is very low.

## 4. Discussion

Here, we describe the construction of a powerful protein-depletion system in *S. pombe* based upon the auxin-inducible degron (AID) system. We created two sets of plasmid vectors for this study. The first is a series of stable integration expression vectors that target the *S. pombe arg3-D4* locus (Figure S1). The vectors include the *arg3* gene sequence that is deleted at the *arg3-D4* locus, a range of *S. pombe adh1* gene promoter sequences of varying strengths and, to clone the gene to be expressed, an MCS. The plasmid vectors are designed to integrate a single stable copy at the *arg3-D4* locus restoring arginine prototrophy.

The second set of plasmids are to fuse the AID tag to the C-terminus of the target protein (Figure 1E). The AID constructs include a flexible ten amino acid linker, the full-length *A. thaliana* IAA17 AID tag (a 229-amino-acid peptide) and, for the detection of AID-tagged proteins by immunoblot, a series of common epitope tags for which commercial antibodies are available. Also included is the option of one of two ‘Determinants of Selective Removal’ (DSR) elements: one derived from the *S. pombe spo5* gene and an improved, mutated version of the *spo5*DSR termed eDSR. DSR elements usually prevent the accumulation of meiosis-specific mRNAs during the mitotic cell cycle in fission yeast by targeting them for destruction by nuclear exosomes. Here, they result in the constitutive reduction in AID-tagged protein levels. The AID constructs can be inserted into the genome using Cre recombinase-mediated cassette exchange [27] or by using a standard PCR-mediated tagging procedure commonly used for fission yeast.

Using the integration vectors, we successfully inserted at the *arg3-D4* locus sequences to express the auxin receptor TIR1 protein from *O. sativa* (OsTIR1^WT^) and an auxin-binding site mutant of OsTIR1, OsTIR1^F74A^. Importantly, both forms of OsTIR1 were not fused to Skp1. Overexpression of either OsTIR1^WT^ or OsTIR1^F74A^ from the strong constitutive *adh1* promoter had no observable effect on cell growth. OsTIR1^F74A^ binds the auxin analog 5′adamantyl-IAA with a higher affinity than OsTIR1 and natural auxin [18]. This synthetic pairing forms the basis of AID version 2 (AID2) the equivalent of which has recently been shown to perform better than AID in chicken DT40 cells, budding yeast, mammalian cells and mice [6,19].

Both AID and AID2 produced a conditional Mcm4 null phenotype at 30 °C. However, as predicted, AID2 required much lower ligand concentration (5,000-fold less), showed slightly faster degradation kinetics and no auxin-independent proteolysis of Mcm4 or Rad52 as compared with AID. The effective concentration of 5-adamaltyl-IAA was higher for Rad52 (100 nM) than for Mcm4 (10 nM). This was surprising considering quantitative analysis of the fission yeast proteomes has shown that in vegetatively growing cells there are approximately 3-fold more Mcm4 protein molecules than Rad52 protein molecules [38] and Nishimura et al. [19] have demonstrated that depletion of AID-tagged CENP-H by 5′-adamantyl-IAA is dose dependent. The AID system may therefore be intrinsically better suited to regulating essential proteins: below a critical threshold level, the cell becomes inviable and a null phenotype achieved. Despite the higher ligand concentration, a lower steady-state protein level and the use of highly effective DSR elements, residual Rad52 activity remains after depletion (Figure 4C) (due possibly to TIR1 being limiting). Tandem arrays of functional truncated forms of the AID tag have been used to dramatically increase the efficiency of previous AID systems (termed ‘3XminiAID’; [39,40]). Use of a 3Xmini-AID-style tag may be sufficient to achieve a conditional-null Rad52 phenotype and we are currently testing this. However, anecdotal evidence from our lab suggests that fusion of the 3Xmini-AID tag to the target protein either partially or completely removes protein function more often than with full-length AID.

Despite no conditional null phenotype for Rad52 being achieved using either AID system, AID2 performed better than AID. The use of DSR elements successfully improved the severity of the conditional mutant phenotype, but a very low level of Rad52 activity remains. Despite the eDSR reducing initial protein levels by 94% and *spo5*DSR by 74% and immunoblots showing less Rad52-AID in *rad52-AID-eDSR* cells treated with 5′adamantyl-IAA than *rad52-AID-spo5DSR* cells treated with 5′adamantyl-IAA, *rad52-AID-eDSR* and *rad52-AID-spo5DSR* cells surprisingly exhibited the same level of HU sensitivity.

A detailed protocol for the synthesis of 5′adamantyl-IAA is provided in the materials and methods section. In addition, 5′adamantyl-IAA is now commercially available (https://www.tcichemicals.com/GB/en/p/A3390 (accessed on 6 June 2021)). We found 5′adamantyl-IAA to be more chemically stable than NAA. We incubated an aliquot of 5′adamantyl-IAA at room temperature for 7 days and, when tested, we observed no loss in activity (Figure S4). In our hands, NAA was found to be chemically labile with freeze/thawing of frozen stocks reducing activity. Only freshly prepared NAA stocks were used.

## 5. Conclusions

Overall, we have significantly improved the utility of the auxin-inducible degron system in *S. pombe* and this should enhance the study of gene function in vivo by more effectively reducing the levels of gene product. As auxin response has been shown to be intrinsically linked to cellular TIR1 concentration [10,32,33], the increase in TIR1 expression level is the probable reason for the concomitant increase in AID efficiency. The relative thermostability of OsTIR1 compared to AtTIR1 [1] also means that our new system works also more efficiently at the higher temperatures of 30 and 36 °C.

The OsTIR system was further enhanced by introducing the synthetic pairing of the binding-site mutant OsTIR1^F74A^ with the auxin analogue 5′adamantyl-IAA (AID2). Because neither the AID or AID2 systems abolish AID-tagged protein levels completely, we also introduced the ability to regulate the initial protein levels by introducing a DSR sequence that restricts the mRNA level and thus reduces the starting protein level. This may be particularly helpful when it is desirable to reduce gene function as much as possible to approach a null phenotype. While both the AID and AID2 systems are highly effective for regulating essential proteins to achieve a null-like phenotype, as seen for Mcm4, we note that residual activity remains when assessing a non-essential gene for function, even when combining AID2 with DSR elements.

## Figures and Tables

**Table 1:** Strains used in this study.

Name	Genotype (*h+*/*h-* Denote Mating Type)
AW279	*h+*
AW1502	*arg3-D4 h+*
AW1581	*rad52::kanMX6 h+*
AW1655	*bleMX6-arg3-D4 h-*
AW1658	*arg3::bleMX6-arg3+-Padh1-OsTIR1^F74A^-TADH1 h-*
AW1660	*arg3::bleMX6-arg3+-Padh1-OsTIR1^WT^-TADH1 h-*
AW1680	*rad52-HA-AID-Turg1:kanMX6, arg3::bleMX6-arg3+-Padh1-OsTIR1^F74A^-TADH1 h-*
AW1681	*rad52-HA-AID-Turg1:kanMX6, arg3::bleMX6-arg3+-Padh1-OsTIR1^F74A^-TADH1, h+*
AW1682	*rad52-HA-AID-spo5DSR-Turg1:kanMX6, arg3::bleMX6-arg3+-Padh1-OsTIR1^F74A^-TADH1, h+*
AW1683	*rad52-HA-AID-spo5DSR-Turg1:kanMX6, arg3::bleMX6-arg3+-Padh1-OsTIR1^F74A^-TADH1, h-*
AW1703	*arg3::bleMX6-arg3+-Padh1-OsTIR1^F74A^-TADH1 h+*
AW1762	*arg3::bleMX6-arg3+-Padh1-OsTIR1^WT^-TADH1 h+*
AW1893	*mcm4-AID-Turg1:kanMX6, h+*
AW1899	*mcm4-AID-V5-Turg1:kanMX6, h+*
AW1901	*rad52-AID-Turg1:kanMX6, h+*
AW1907	*rad52-AID-V5-Turg1:kanMX6, h+*
AW1915	*rad52-AID-spo5DSR-Turg1:kanMX6 h+*
AW1921	*rad52-AID-V5-spo5DSR-Turg1:kanMX6 h+*
AW1923	*mcm4-AID-Turg1:kanMX6, arg3::bleMX6-arg3+-Padh1-OsTIR1^WT^-TADH1 h+*
AW1925	*mcm4-Turg1:kanMX6, arg3::bleMX6-arg3+-Padh1-OsTIR1^F74A^-TADH1 h+*
AW1935	*mcm4-AID-V5-Turg1:kanMX6, arg3::bleMX6-arg3+-Padh1-OsTIR1^WT^-TADH1 h+*
AW1937	*mcm4-AID-V5-Turg1:kanMX6, arg3::bleMX6-arg3+-Padh1-OsTIR1^F74A^-TADH1 h+*
AW1942	*ade6::ade6+-Padh15-skp1-AtTIR1-2NLS-9myc h+*
AW1949	*mcm4-AID-Turg1:kanMX6, ade6::ade6+-Padh15-skp1-AtTIR1-2NLS-9myc h+*
AW1962	*rad52-AID-Turg1:kanMX6, arg3::bleMX6-arg3+-Padh1-OsTIR1^F74A^-TADH1 h+*
AW1968	*rad52-AID-V5-Turg1:kanMX6, arg3::bleMX6-arg3+-Padh1-OsTIR1^F74A^-TADH1 h+*
AW1970	*rad52-AID-spo5DSR-Turg1:kanMX6, arg3::bleMX6-arg3+-Padh1-OsTIR1^F74A^-TADH1 h+*
AW1976	*rad52-AID-V5-spo5DSR-Turg1:kanMX6, arg3::bleMX6-arg3+-Padh1-OsTIR1^F74A^-TADH1 h+*
AW1990	*rad52-AID-Turg1:kanMX6, arg3::bleMX6-arg3+-Padh1-OsTIR1^WT^-TADH1 h+*
AW1996	*rad52-AID-V5-Turg1:kanMX6, arg3::bleMX6-arg3+-Padh1-OsTIR1^WT^-TADH1 h+*
AW2011	*rad52-AID-eDSR-Turg1:kanMX6 h+*
AW2017	*rad52-AID-V5-eDSR-Turg1:kanMX6 h+*
AW2024	*rad52-AID-eDSR-Turg1:kanMX6, arg3::bleMX6-arg3+-Padh1-OsTIR1^F74A^-TADH1 h+*
AW2026	*rad52-AID-V5-eDSR-Turg1:kanMX6, arg3::bleMX6-arg3+-Padh1-OsTIR1^F74A^-TADH1 h+*

**Table 2:** Sequences of oligonucleotide primers used in this study.

Name	Sequence (5′ to 3′)
P1	AGAAATCTATAGAAAAAAAGCTAGCGTGACGCAGACA
P2	CTCCCGGGAGTGCATGCCAGCATATGTATGTGGTTAGAAAAAAGAAAAGACTTAAAAG
P3	AGAAATCTATAGAAAAAAAGCTAGCGTGACGCAGACATTCGAATGGCATGCCC
P4	CTCCCGGGAGTGCATGCCAGCATATGTATGTGGTTAGAAAAAAGAAAAGACTTAAAAGTTTGTGATAGTCAAGACAATGGAATTCTCTTGCTTAAAGAAAAGCGAAGGCA
P5	GAGAGCTCCGTCTGCAGCGAGTCGACACTTCTAAATAAGCGAATTTCTTATGATTTATGA
P6	ACACTCTACTTGCCCAGATCACTAGTATATTACCCTGTTATCCCTAGCG
P7	CATACATTATACGAAGTTATGCATGCTCGGTGGGTCAGGTGGAAGTGGATCTGGTGCTATGATGGGCAGTGTCGA
P8	AAATTCGCTTATTTAGAAGTGCTAGCTCAAGCTCTGCTCTTGCACTTC
P9	AGTGCAAGAGCAGAGCTTGAGCTAGCAGTCCCGGGGCTTGCCCATCTGTTTTAGACGT
P10	GGGGACGAGGCAAGCTAAACAGATCTCGAGAAGAAGGCCCCGCTG
P11	AGTGCAAGAGCAGAGCTTGAGCTAGCACTACGCCATATCATGCCCA
P12	TCTAAAACAGATGGGCAAGCCCCGGGGCTTTGTCTAACAGGTTTTATGTTGGTTTAAGT
P13	AGAAGTGCAAGAGCAGAGCTGGTGCTGGAGCAGGCGCCTACCCATACGATGTTCCTGACTATGCG
P14	GATGGGCAAGCCCCGGGACTGCTAGCGGCGCGCCTCAGCACTGAGC
P15	TGGGCATGATATGGCGTAGTGCTAGCGGCGCGCCTCAGCACTGAGC
P16	AGAAGTGCAAGAGCAGAGCTGGAGCAGGCGCCGGTGAACAAAAGTTGATTTCTGAAG
P17	GATGGGCAAGCCCCGGGACTGCTAGCGGCGCGCCTTACAAGTCTTCCTC
P18	TGGGCATGATATGGCGTAGTGCTAGCGGCGCGCCTTACAAGTCTTCCTC
P19	TATACATTATTTAATACTAGTCCTAACTGACACAGTACAATATTCATTATTTCTATGCAAGCCAGTTCAATATCTTGATTGTTTAGCTTGCCTCGTCCCC
P20	TCATCAGGGTCATTGGGACTATTCAACGCGAAATCAAATAGAAAACAAAATATTGTTTCAAAAGAATGCTTTCATGTATAGGATGGCGGCGTTAGTATCG
P21	GTGCTTTGGAAAGGCGAGGACGTATTAAGGTTATTACCAGTGCTGGACATCGCATTGTACGTTCAATTGCACAGACTGATGGTGGGTCAGGTGGAAGTG
P22	ATTATGCTCTGTAGTCTTTGATTTTCAACAACGACCTATGGTTTATGGCTCATGAATAATACCAGCTTATTCGCTAAAAAAGGATGGCGGCGTTAGTATC
P23	GAACAAATTCTGATCCTCAGTCGGCAATGAGGTCGCGAGAAAACTACGATGCTACGGTGGATAAGAAAGCCAAAAAAGGAGGTGGGTCAGGTGGAAGTG
P24	AGATCTACCGTTTAAACAAATCATTAGTCATAAAACAGAAAATACTTGGTAAAAAACAAGTTGCCAATCATCACATTTTGCCTCATTACTTGGATGGCGGCGTTAGTATC

## Data Availability

Primary data are available from the authors on request.

## Supplementary Materials

The following are available online at https://www.mdpi.com/article/10.3390/genes12060882/s1, Figure S1: Overview of the *arg3-D4* integration expression vector system. (a) Sequence of the *arg3-D4* locus [24]. Underlined capital letters: pBR322 plasmid sequence; sequence highlighted in yellow: *arg3-D4* homology used in construction of *arg3+* expression vectors (see b). (b) Schematic illustration showing *arg3-D4* integration expression vectors. HOM: the two homology sequences used to insert the linear expression construct at the *arg3-D4* locus (see a); MCS: multiple clone site; number in parenthesis after plasmid name: Addgene ID. (c) Schematic illustration showing integration of *P_adh1_-OsTIR1^WT^-NLS-T_ADH1_* and *P_adh1_-OsTIR1^F74A^-NLS-T_ADH1_* expression constructs at *arg3-D4* locus using *arg3-D4* integration expression vector system; *cmb1*: gene neighbouring *arg3*. Figure S2: The *spo5*DSR and eDSR elements. (a) The 157bp DSR element from the *S. pombe spo5* gene downstream region as identified by Harigaya et al. [20]. Sequences identical to core hexanucleotide U(U/C)AAAC motifs are highlighted. Boxed sequences are those identified as similar to the core motif. (b) The 158 bp eDSR sequence derived from the *spo5*DSR element. Underlined sequences are additional core TTAAAC motifs (mutated bases shown in lower case). Figure S3: Alignment of *Oryza sativa* transport inhibitor response protein 1 (OsTIR1) and *Arabidopsis thaliana* transport inhibitor response protein 1 (AtTIR1) proteins. AtTIR1 (GeneID 825473) and OsTIR1 (GeneID 4335696) were aligned using the ClustalW software. Black and grey boxes indicate identical and similar amino acids, respectively. Position F79 of AtTIR1 and the corresponding F74 of OsTIR1 are arrowed. Figure S4: 5’adamantyl-IAA is chemically stable. Isogenic strains with indicated genotypes (*rad52+* (AW279), *rad52-HA-AID, OsTIR1^F74A^* (AW1680), *rad52-HA-AID OsTIR1^F74A^* (AW1681), *rad52-HA-AID-spo5DSR OsTIR1^F74A^* (AW1682), *rad52-HA-AID-spo5DSR OsTIR1^F74A^* (AW1683), *rad52*Δ (AW1581)) were spotted onto YEA plates and plates containing 100 nM 5’adamantyl-IAA (5’a-IAA) and/or genotoxic agent hydroxyurea (HU) at the indicated concentrations. 5’adamantyl-IAA added to plates from frozen stock (−20 °C stock) or from an aliquot incubated at room temperature for 7 days (7d RT). Plates were grown at 30 °C for 3 days, Figure S5: Titration of 5’adamantyl-IAA to determine effective ligand concentration. (a) Mcm4-AID. Isogenic strains with indicated genotypes (*mcm4+* (AW279), *mcm4-AID* (AW1893), *OsTIR1^F74A^* (AW1703), *mcm4-AID OsTIR1^F74A^* (AW1925)) were spotted onto YEA plates and plates containing 100 nM 5’adamantyl-IAA (5’a-IAA), 10 nM 5’adamantyl-IAA (5’a-IAA) and 1 nM 5’adamantyl-IAA (5’a-IAA). Plates were grown at 30 °C for 3 days. (b) Rad52-AID. Isogenic strains with indicated genotypes (*rad52+* (AW279), *rad52-AID* (AW1901), *OsTIR1^F74A^* (AW1703), *rad52-AID OsTIR1^F74A^* (AW1962), *rad52*Δ (AW1581)) were spotted onto YEA plates and plates containing 1000 nM 5’adamantyl-IAA (5’a-IAA), 100 nM 5’adamantyl-IAA (5’a-IAA) and 10 nM 5’adamantyl-IAA (5’a-IAA). Plates were grown at 30 °C for 3 days. Note: each panel in (b) is a composite of two images (both taken of the same plate) excising irrelevant strains between *rad52-AID* and *rad52-delete*.
