# Supplementary material for: Rice (Oryza sativa) TIR1 and 5′adamantyl-IAA Significantly Improve the Auxin-Inducible Degron System in Schizosaccharomyces pombe
Source: Genes (Basel). 2021 Jun 8;12(6):882. doi: 10.3390/genes12060882 (PMC8229956; doi:10.3390/genes12060882)
Supplement: Supplementary file 1 [file genes-12-00882-s001.zip › genes-1231440-supplementary.pdf]

genes

MDPI

Fig S1

(A)

```
agcacttatcataaaatttattatctaagataatctcgttaaaaaaaaaaaaaaaaaaaaaaaaaaaaaaaatttggcaagaagc
ccttctctttcgttaaaacttgtttcgctttatgcaatactgtaagtgtaagcttgtaatatagcgaatctgtgaactcattgtt
catttcatggtagttgttgatttcacaagcatgctgagttatcaatatagtaacacaattggttttatttcagctcgcttgaata
tagtgtcaagagataattgaataaacaaaaaaaataactacacgtttttttagatacattttatttacaatgtattggttctttaa
agaatgggtggtaaataaaaaaaatattacaaggtcgaaaaaaattcatcccttttcattagttctataattaaatgctaaaccc
atgaatattcttttactgctaatcaattaacgattttggtatttaaaagctattgctgataagtattctgacgttctgtatcaat
attaccatgaataacataagaaaaagaaaggaaatagaatgtttatgagtagtttttaatttttgacttgttttatcatatttca
tcaacgtactagcttgtttgcaactgaTAGCTTTAATGCGGTAGTTTATCACAGTTAAATTGCTAACGCAGTCAGGCACCGTGTA
TGAAATCTAACAATGCGCTCATCGTCATCCTCGGCACCGTCACCCTGGATGCTGTAGGCATAGGCTTGGTTATGCCGGTACTGCC
GGGCCTCTTGCGGGATATCGTCCATTCCGACAGCATCGCCAGTCACTATGGCGTGCTGCTAGCGCTATATGCGTTGATGCAATTT
CTATGCGCACCCGTTCTCGGAGCACTGTCCGACCGCTTTGGCCGCCGCCCAGTCCTGCTCGCTTCGCTACTTGGAGCCACTATCG
ACTACGCGATCATGGCGACCACACCCGTCCTGTGgatctgggcaagtagagtgtgcatcatcgaaatccggcttctttcataaac
ttatcgtattgctctttaagtgctttgcttttatcaataaatggctttttcttgtcttccgccaattccttccacgcctttgatg
cattttgggtctcttcaacgagtgtggaaatggcttctggaatacctaactcttgacgtagcttgggattgtttcgaagctcctt
gtaaaagaggataaaagcagaagaaggtttttaggaacatctggatgacgaagtctgtaatagcgactgcccgtgctgtgagcc
tccttcgagcgacgacgattttcactatcgatttctgtaggtgtaaaggatcgtagagtctccataaaacgctttttctcagcat
cgaactgctcctggagtttgtttttatactttggatctcatcggcagtcaaagctttgtatttttgacgaatcatctcaaagcg
ctcaggaccattaccagcaccttttgtttcaaccaacatttgattccaaatggttttcagcctagggggatcaacttctgtaat
```

(B)

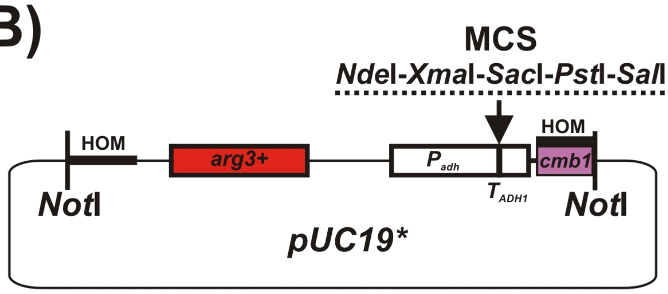

P_{adh1}-MCS-T_{ADH1}-Sp.arg3+/pUC19* (169349)

P_{adh15}-MCS-T_{ADH1}-Sp.arg3+/pUC19* (169350)

P_{adh81}-MCS-T_{ADH1}-Sp.arg3+/pUC19* (169351)

(C)

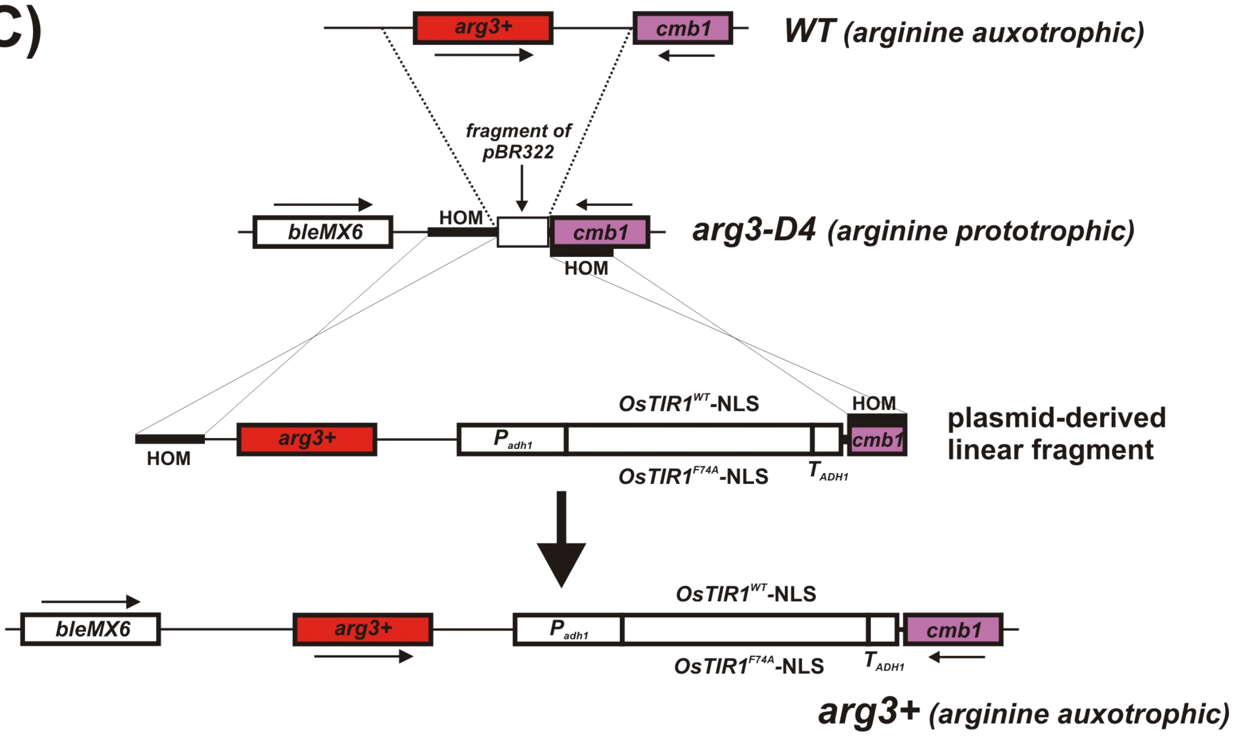

Figure S1. Overview of the *arg3-D4* integration expression vector system. (**a**) Sequence of the *arg3-D4* locus [24]. Underlined capital letters: pBR322 plasmid sequence, sequence highlighted in yellow: *arg3-D4* homology used in construction of *arg3+* expression vectors (see b). (**b**) Schematic illustration showing *arg3-D4* integration expression vectors. HOM: the two homology sequences used to insert the linear expression construct at the *arg3-D4* locus (see a), MCS: multiple clone site, number in parenthesis after plasmid name: Addgene ID. (**c**) Schematic illustration showing integration of P_{adh1}-$OsTIR1^{WT}$-NLS-T_{ADH1} and P_{adh1}-$OsTIR1^{F74A}$-NLS-T_{ADH1} expression constructs at *arg3-D4* locus using *arg3-D4* integration expression vector system *cmb1*: gene neighbouring *arg3*.

S2

(A)

spo5DSR (157bp)

```
ACTACGCCATATCATGCCCATCCCGCTACTTCTGCTGCTAATTAAATTTAAACATCTTTCCAGCATTTACTTGAACA
TCAAACTCTAATATCGAAATCTTTTCAAACACTTATCACATACTCAAACTTAAACCAACATAAAACCTGTTAGACAA
AGC
```

(B)

eDSR (158bp)

```
ACTACGCCATATCATGCCCATCCCGCTACTTCTGCTGCTAATTAAAcTTTAAACATCTTTCCAGCATTTACTTGAACA
TCAAACTCTAATATCGAAATCTTTTCAAACACTTATCACATACTCAAACTTAAACCAACATtAAACCTGTTAGACAAA
AGC
```

Figure S2. The *spo5*DSR and eDSR elements. (**a**) The 157bp DSR element from the *S. pombe spo5* gene downstream region as identified by Harigaya et al. [20]. Sequences identical to core hexanucleotide U(U/C)AAAC motifs are highlighted. Boxed sequences are those identified as similar to the core motif. (**b**) The 158bp eDSR sequence derived from the *spo5*DSR element. Underlined sequences are additional core TTAAAC motifs (mutated bases shown in lower case).

S3

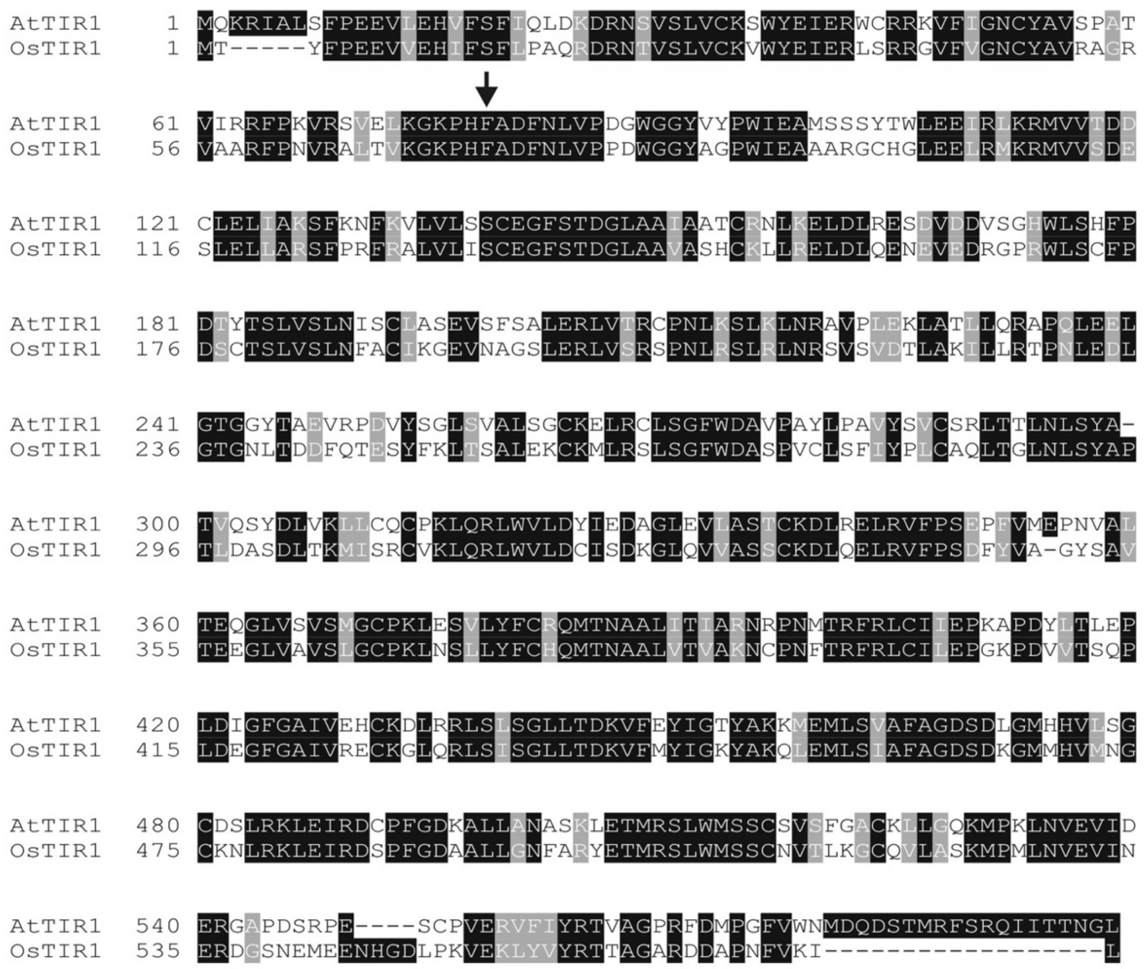

Figure S3. Alignment of *Oryza sativa* transport inhibitor response protein 1 (OsTIR1) and *Arabidopsis thaliana* transport inhibitor response protein 1 (AtTIR1) proteins. AtTIR1 (GeneID 825473) and OsTIR1 (GeneID 4335696) were aligned using the ClustalW software. Black and grey boxes indicate identical and similar amino acids, respectively. Position F79 of AtTIR1 and the corresponding F74 of OsTIR1 are arrowed.

Fig S4

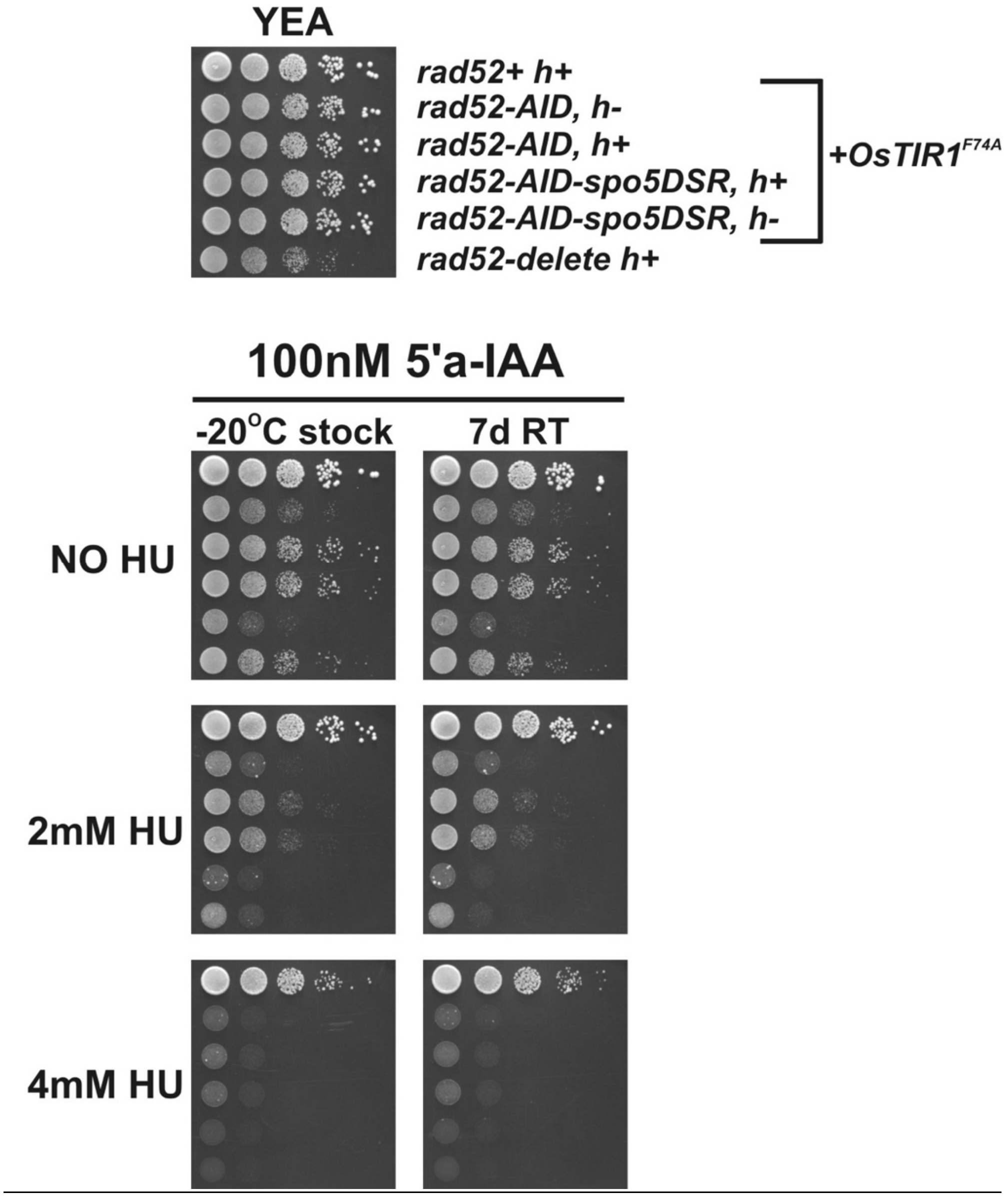

Figure S4. 5'adamantyl-IAA is chemically stable. Isogenic strains with indicated genotypes (*rad52+* (AW279), *rad52-HA-AID, OsTIR1^{F74A}* (AW1680), *rad52-HA-AID OsTIR1^{F74A}* (AW1681), *rad52-HA-AID-spo5DSR OsTIR1^{F74A}* (AW1682), *rad52-HA-AID-spo5DSR OsTIR1^{F74A}* (AW1683), *rad52*Δ (AW1581)) were spotted onto YEA plates and plates containing 100nM 5'adamantyl-IAA (5'a-IAA) and/or genotoxic agent hydroxyurea (HU) at the indicated concentrations. 5'adamantyl-IAA added to plates from frozen stock (-20°C stock) or from an aliquot incubated at room temperature for 7 days (7d RT). Plates were grown at 30°C for 3 days.

Fig S5

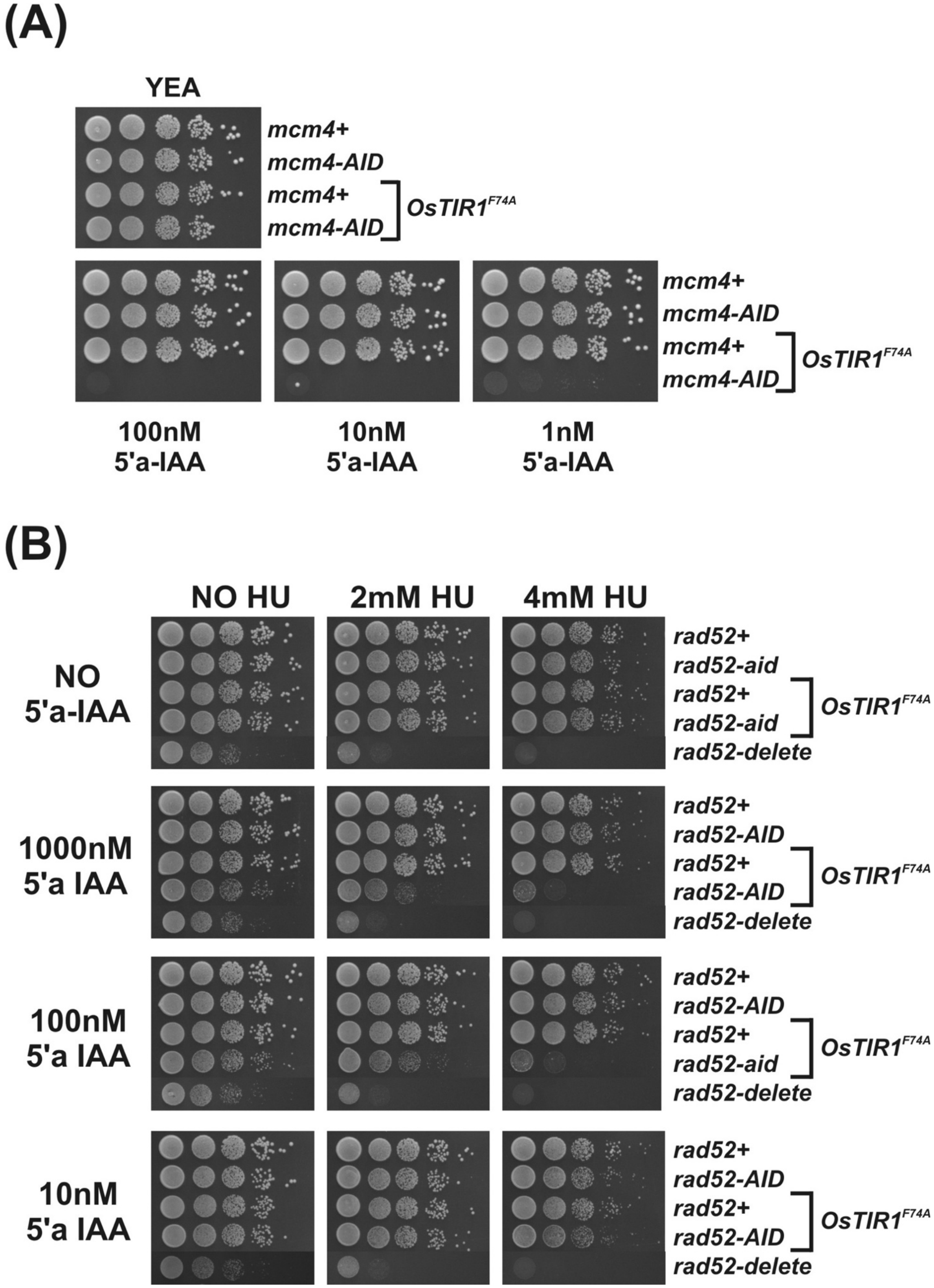
(A)
YEA
mcm4+
mcm4-AID
mcm4+
mcm4-AID
OsTIR1F74A
100nM 5'a-IAA
10nM 5'a-IAA
1nM 5'a-IAA
(B)
NO HU
2mM HU
4mM HU
NO 5'a-IAA
1000nM 5'a IAA
100nM 5'a IAA
10nM 5'a IAA
rad52+
rad52-aid
rad52-AID
rad52-delete
OsTIR1F74A

Figure S5. Titration of 5'adamantyl-IAA to determine effective ligand concentration. (**a**) Mcm4-AID. Isogenic strains with indicated genotypes (*mcm4+* (AW279), *mcm4-AID* (AW1893), *OsTIR1*^{F74A} (AW1703), *mcm4-AID OsTIR1*^{F74A} (AW1925)) were spotted onto YEA plates and plates containing 100nM 5'adamantyl-IAA (5'a-IAA), 10nM 5'adamantyl-IAA (5'a-IAA)and 1nM 5'adamantyl-IAA (5'a-IAA). Plates were grown at 30°C for 3 days. (**b**) Rad52-AID. Isogenic strains with indicated genotypes (*rad52+* (AW279), *rad52-AID* (AW1901), *OsTIR1*^{F74A} (AW1703), *rad52-AID OsTIR1*^{F74A} (AW1962), *rad52*Δ (AW1581)) were spotted onto YEA plates and plates containing 1000nM 5'adamantyl-IAA (5'a-IAA), 100nM 5'adamantyl-IAA (5'a-IAA) and 10nM 5'adamantyl-IAA (5'a-IAA). Plates were grown at 30°C for 3 days. Note: each panel in (b) is a composite of two images (both taken of the same plate) excising irrelevant strains between *rad52-AID* and *rad52-delete*.
